# In Vivo T-Box Transcription Factor Profiling Reveals Joint Regulation of Embryonic Neuromesodermal Bipotency

**DOI:** 10.1016/j.celrep.2013.08.012

**Published:** 2013-09-26

**Authors:** George E. Gentsch, Nick D.L. Owens, Stephen R. Martin, Paul Piccinelli, Tiago Faial, Matthew W.B. Trotter, Michael J. Gilchrist, James C. Smith

**Affiliations:** 1 Division of Systems Biology, National Institute for Medical Research, London NW7 1AA, UK; 2 Wellcome Trust/Cancer Research UK Gurdon Institute, Cambridge CB2 1QN, UK; 3 Department of Zoology, University of Cambridge, Cambridge CB2 3EJ, UK; 4 Division of Physical Biochemistry, National Institute for Medical Research, London NW7 1AA, UK; 5 Anne McLaren Laboratory for Regenerative Medicine, Cambridge CB2 0SZ, UK

## Abstract

The design of effective cell replacement therapies requires detailed knowledge of how embryonic stem cells form primary tissues, such as mesoderm or neurectoderm that later become skeletal muscle or nervous system. Members of the T-box transcription factor family are key in the formation of these primary tissues, but their underlying molecular activities are poorly understood. Here, we define in vivo genome-wide regulatory inputs of the T-box proteins Brachyury, Eomesodermin, and VegT, which together maintain neuromesodermal stem cells and determine their bipotential fates in frog embryos. These T-box proteins are all recruited to the same genomic recognition sites, from where they activate genes involved in stem cell maintenance and mesoderm formation while repressing neurogenic genes. Consequently, their loss causes embryos to form an oversized neural tube with no mesodermal derivatives. This collaboration between T-box family members thus ensures the continuous formation of correctly proportioned neural and mesodermal tissues in vertebrate embryos during axial elongation.

## Highlights

• The T-box factors Eomes, VegT, and Xbra largely bind the same DNA binding sites
• They control stem cell differentiation into neural or mesodermal tissue in vivo
• Joint loss of T-box factors entirely prevents mesoderm formation in vertebrate embryos
• Promoter-proximal T-box factors recruit RNA Pol II for transcriptional activation

## Graphical Abstract

**Figure:**
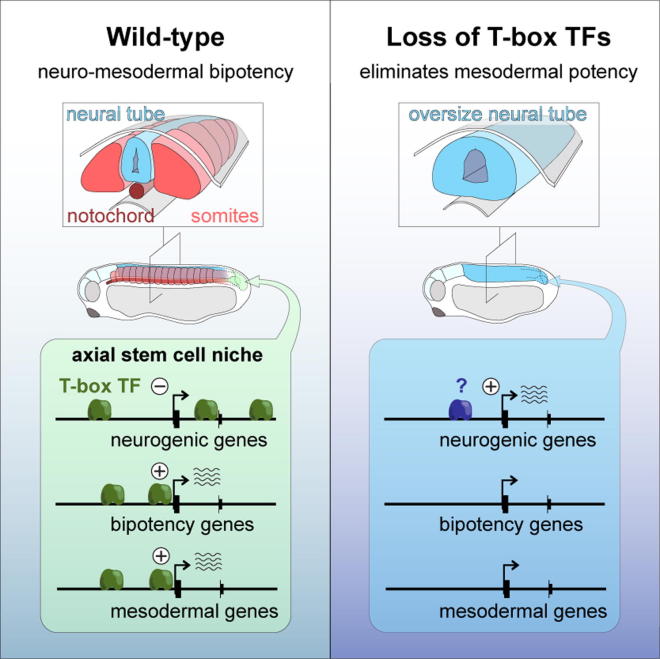

## Teaser

The development of effective cell replacement therapies requires detailed knowledge of how embryonic stem cells form primary tissues, such as mesoderm or neurectoderm that later become skeletal muscle or spinal cord. Gentsch, Smith, and colleagues now provide mechanistic insight into how T-box transcription factors regulate stem cells to form neural or mesodermal tissues. The authors show how this ensures the harmonious formation of spinal cord, muscle, and notochord as the vertebrate embryo elongates along its anteroposterior axis.

## Introduction

As the vertebrate embryo elongates along its anteroposterior axis, primary tissues are produced in a continuous fashion to form trunk and tail. This process is thought to occur as a continuation of gastrulation, during which period primary tissues, such as neurectoderm and mesoderm, emerge for the first time. Recent research concluded that axial elongation is driven mainly by neuromesodermal stem cells at the caudal end of the embryo that go on to form the posterior nervous system and mesodermal derivatives, such as skeletal muscle and notochord (Davis and Kirschner, 2000; Gont et al., 1993; Tzouanacou et al., 2009). Prominent among the genes that influence the fate of early embryonic cells are members of the T-box transcription factor (TF) family, including *Brachyury* (also known as *T*), *Eomesodermin* (*Eomes*), and *VegT*. For example, mouse embryos that lack *Brachyury* fail to form mesoderm posterior to somites 8–12 (Chesley, 1935). Previous analyses of *Brachyury* (*Xbra*), *Eomes*, and *VegT* in the *Xenopus* embryo have focused on their expression patterns, their powerful transactivation activities, and their ability to cause isolated ectodermal tissue to activate mesoderm-specific genes (Showell et al., 2004). However, the way in which T-box TFs exert such profound effects in vertebrate embryos during normal development remains poorly understood.

By combining genome-wide chromatin profiling, gain- and loss-of-function experiments, and quantification of DNA binding dynamics, we now provide mechanistic insights into the T-box-mediated cell fate switches that cause neural and mesodermal tissues to form in the correct proportions along the rostrocaudal axis of the vertebrate embryo. Mesodermal cell fate is defined by multiple T-box TFs, and their combined loss restricts the fates of neuromesodermal stem cells such that the embryo forms excess neural tissue at the expense of mesoderm.

## Results

### Xbra Is Stably Recruited to Motif Variants in Early Development

**Figure 1:**
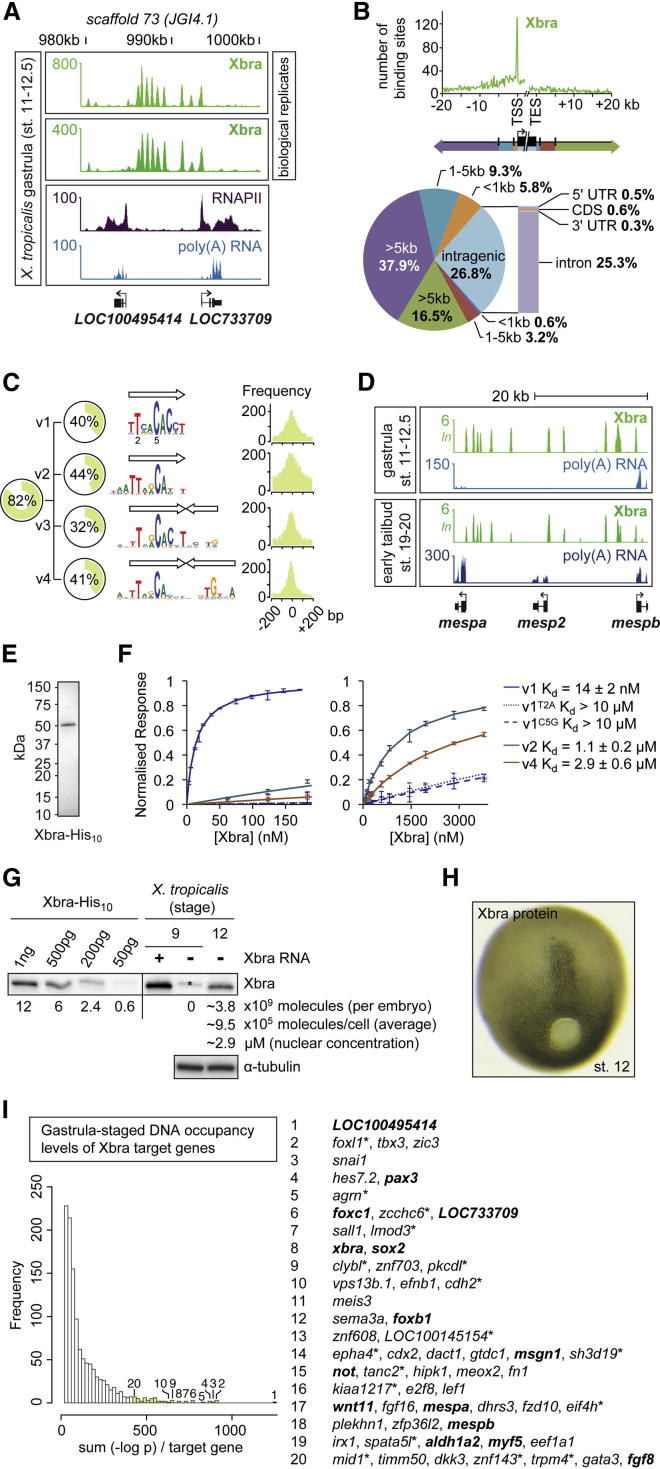
Xbra Is Stably Recruited to Mono- and Dimeric Motif Variants in the *X. tropicalis* Genome during Early Embryogenesis (A) Excerpt of normalized Xbra binding at gastrula stage. RNAPII and poly(A) RNA profile from Akkers et al. (2009). (B) Genomic distribution of Xbra binding sites (FDR ≤ 1%) relative to the start (TSS) and end (TES) of transcription of nearest target genes. (C) De novo motif discovery analysis of Xbra-bound regions with coverage, sequence logo, and positional distribution for each T-box motif variant (v1–v4). Arrow indicates monomeric binding site. (D) Comparison of Xbra binding at gastrula and early tail bud stage near *mesp* gene cluster with poly(A) RNA profile from Akkers et al. (2009) and this study. (E) Coomassie staining of 0.5 μg purified Xbra-His_10_ run on a SDS-polyacrylamide gel. (F) Surface plasmon resonance diagrams (normalized response versus Xbra concentration) including K_d_ values for the interaction between native Xbra protein and different DNA motifs (v1, v2, and v4). Superscript T2A and C5G refer to base changes introduced in v1. (G) Quantification of Xbra protein levels in midgastrula embryos (stage 12) by western blotting with standard curve of purified Xbra-His_10_ as indicated. Positive control, pregastrula embryo (stage 9) injected with RNA encoding untagged Xbra. Protein extracts equivalent to two embryos at stages 9 (negative control) and 12 were loaded. Asterisk marks nonspecific band seen at stage 9. The same band is present at the same intensity in the absence of Xbra at stage 12 (data not shown), and its intensity was therefore subtracted from the Xbra band for quantification. Further calculations (molecules/cell) and nuclear concentrations (μM) are based on an estimated 4,000 Xbra-positive cells at stage 12 ([H]; Cooke, 1979), a nuclear envelope (sphere) surface of ∼300 μm^2^ (Levy and Heald, 2010), and 90% of Xbra being nuclear. Loading control, α-tubulin. (H) Whole-mount immunohistochemistry of Xbra protein in a midgastrula embryo (stage 12). (I) Histogram of nearest gene-associated Xbra binding levels as detected by ChIP-seq. The asterisk indicates genes with nearest Xbra binding >10 kb from TSS. Genes in bold are mentioned elsewhere in this study. See also Figure S1 and Table S1.

**Figure S1:**
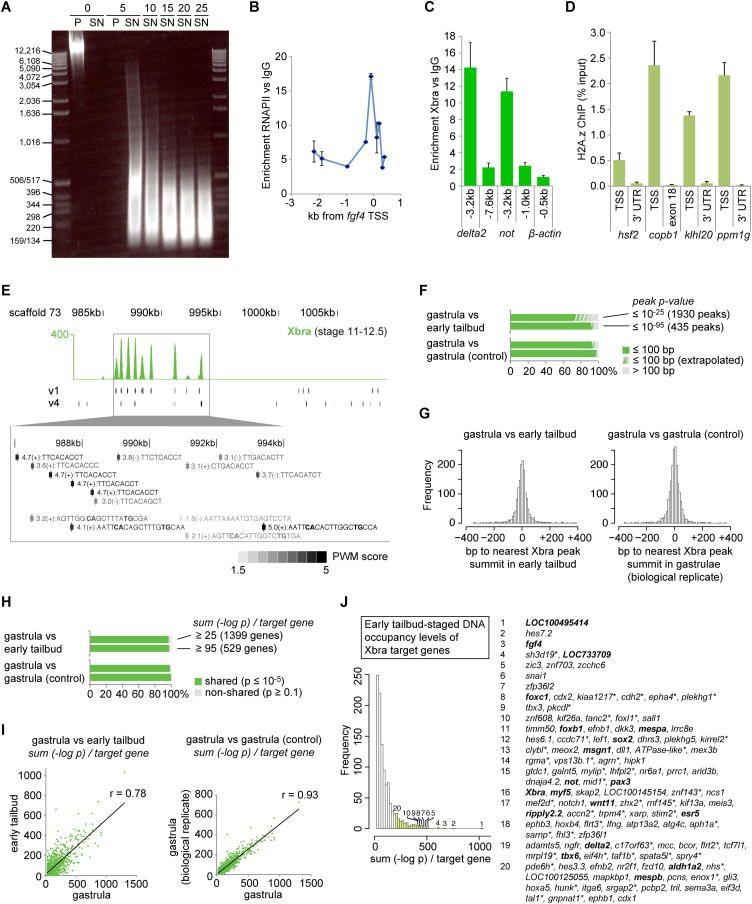
Development of the ChIP Protocol for *Xenopus* Embryos Suitable for Deep Sequencing and Near-Identical Xbra Binding Characteristics at Gastrula and Early Tail Bud Stage, Related to Figure 1 (A) Increasing rounds (5, 10, 15, 20 and 25) of sonication caused cross-linked chromatin extracted from X. *tropicalis* gastrula embryos (stage 12) to be solubilised (compare pellet [P] to supernatant [SN] before and after 5 rounds of sonication) and sheared to fragments of the indicated sizes. Ethidium Bromide staining of genomic DNA size-separated by gel electrophoresis. (B) RNA polymerase II (RNAPII) ChIP-qPCR on *X. laevis* gastrula embryos (stage 12) showed RNAPII recruitment to the TSS of *fgf4* (also known as *eFGF(i)* promoter, AF078081) (Casey et al., 1998). DNA enrichment of RNAPII versus IgG ChIP plotted against distance (kb) to transcription start site (TSS) of *fgf4*. Error bars, s.d. of biological duplicates. (C) Xbra ChIP-qPCR on *X. tropicalis* gastrula embryos (stage 12) showed Xbra binding to 3.2-kb upstream region of the *delta2* and *notochord (not)* gene (10- to 15-fold DNA enrichment of Xbra versus IgG ChIP). *not*, *delta2* and *β-actin* (negative control) loci were used as the ‘gold-standard’ controls to improve the efficiency and signal-to-noise ratio of the Xbra ChIP on *X. tropicalis* embryos. Positive control regions were selected based on the homology to respective target genes determined by ChIP on zebrafish mid-gastrula embryos (Morley et al., 2009) and the availability of canonical Xbra recognition sites determined in vitro (Conlon et al., 2001). Error bars, s.d. of biological replicates (n = 2-4). (D) The ChIP protocol devised for *Xenopus* embryos was also applicable to cross-linked zebrafish embryos. H2A.z ChIP-qPCR showed significantly more DNA occupancy (plotted as percentage of DNA input) of the histone variant H2A.z at the TSS (proximal promoter) than at the end (3′ UTR or last exon) of transcriptionally active genes (*hsf2*, *copb1*, *klhl20* and *ppm1g*) in zebrafish bud-staged embryos. Error bars, s.d. of technical triplicates. This binding profile is in accordance to findings reported for the human genome (Barski et al., 2007). (E) Illustration of overlap between Xbra binding as shown in Figure 1A and occurrence of motif variant v1 and v4 (Figure 1C). A magnification of the intergenic region between LOC100495414 and LOC733709 shows the sequences found on the + or - strand that match the position weight matrix (PWM) of v1 and v4 with the indicated score. (F) Co-localization (%) of peak summits (distance ≤ 100 bp) between gastrula and early tailbud stage under conditions of high stringency (p ≤ 10^−25^ or 10^−95^). Extrapolation was based on visual inspection of Xbra binding profiles (see (H)). The comparison of biological replicates of gastrula-specific Xbra binding profile was used as a control in (F)-(I). (G) Histogram of distances between corresponding Xbra peaks (FDR ≤ 1%) detected in gastrula and early tailbud embryos. (H) Overlap (%) of Xbra target genes between gastrula and early tailbud stages under the condition that the sum of Xbra binding exceeds 25 or 95 [-log p] per gene at gastrula or early tailbud stage. Visual inspection of Xbra binding profiles revealed that 44 of the top 50 of non-shared target genes sorted by the sum [-log p] were incorrectly declared as the peak caller failed to identify existent peaks (p ≤ 0.1). The same inspections were carried out for non-shared target genes at tailbud stage (34/40 incorrect) and non-shared target genes of biological replicates (21/45 incorrect). (I) Linear correlation of DNA occupancy levels (sum [-log p]) at individual target genes shared between gastrula and early tailbud embryos. Deviation from this linear positive correlation is reflected by a decreasing Pearson’s correlation coefficient r. (J) Histogram (bins of 20 [-log p]) of nearest gene-associated Xbra binding levels as detected by ChIP-Seq at early tailbud stage (sum [-log p] ≥ 25). ^∗^ indicates genes with nearest Xbra binding further than 10 kb away from TSS. Identified target genes in bold are mentioned elsewhere in this study.

To discover how T-box TFs regulate primary tissue formation in vivo, a genome-wide binding map was first created for Xbra in *X. tropicalis* gastrula embryos (stages 11–12.5) by chromatin immunoprecipitation coupled to deep sequencing (ChIP-seq) (Figure 1A). A protocol (Extended Experimental Procedures) was developed to efficiently extract and shear chromatin from crosslinked *Xenopus* and zebrafish embryos (Figures S1A–S1D). During gastrulation, *Xbra* expression occurs predominantly in nascent mesoderm and in the forming notochord (Figures 4A and S5A). Peak calling (false discovery rate [FDR] ≤ 1%) identified ∼5,500 Xbra binding sites (Table S1) across the nearly fully sequenced (∼88%) genome of *X. tropicalis* (JGI4.1). More than half of Xbra binding was detected upstream of ∼2,700 Ensembl genes, determined according to their shortest distances from Xbra binding sites (Figure 1B). However, a significant number of genes showed binding at lower rather than higher levels (Figure 1I), suggesting that many of them are not regulated by this TF in a way that achieves biological relevance (Biggin, 2011). Most Xbra binding occurred within 400 bp of the transcription start site (TSS), with more than a quarter within gene bodies, mostly in introns (Figure 1B). A de novo search for enriched motifs at Xbra binding sites identified four related motif variants (v1–v4), which, with some overlapping coverage, together account for 82% of binding sites detected at the gastrula stage, suggesting that they are involved in Xbra binding (Figure 1C). However, we note that some peaks do not include any of these variants and that many recognition sites elsewhere in the genome are not occupied by Xbra (Figure S1E). This suggests that Xbra binding in vivo is influenced by other factors, such as tethered binding to other proteins and chromatin accessibility (Neph et al., 2012). The 9 bp motif v1 resembles the consensus sequence TVWCACCH selected by Xbra in vitro (Conlon et al., 2001), but, like motifs v2–v4, it includes an additional 5′ thymine that is likely to make hydrophobic contact with a loop of the Xbra T-domain (Müller and Herrmann, 1997). All discovered motif variants include an adenine preceded by a cytosine, with the corresponding guanine being the main contact point for the T-domain in the major groove of dsDNA (Müller and Herrmann, 1997). Motif v2 retains a strong preference for the initial pair of thymines of v1 and the cytosine followed by an adenine, whereas v3 and v4 comprise partial and almost complete palindromes. Motifs v3 and v4 are more degenerate than v1 and v2, except for the main contact bases of the T-box motif.

The affinities between native full-length Xbra protein (Figure 1E) and DNA motifs v1, v2, and v4 were confirmed in vitro by surface plasmon resonance (Figure 1F). Motif variant v1 showed the strongest affinity for Xbra, with half of the available sites occupied at an Xbra concentration of ∼14 nM (the dissociation constant, K_d_). Base changes at the most strongly conserved positions 2 (alanine for thymine) or 5 (guanine for cytosine) of v1 caused this affinity to drop ∼1,000-fold. The average nuclear concentration of Xbra at the midgastrula stage was quantified as ∼2.9 μM (Figures 1G and 1H). This is 200 times greater than the K_d_ of v1, suggesting that the great majority of accessible v1 motifs are likely to be bound at any time. Motifs v2 and v4 show lower affinities for Xbra of ∼1.1 μM and ∼2.9 μM, respectively, suggesting that their occupancies are more sensitive to changes in Xbra concentration. However, it cannot be excluded that Xbra-associating proteins further influence the stability of these interactions in vivo.

The gastrula-stage genome-wide Xbra binding profile was then compared with that of early tail bud embryos at stages 19 and 20, when *Xbra* expression is confined to the notochord and the caudal end of the embryo (Figure 4A). This comparison indicated that at least 94% of the Xbra binding sites are maintained (overlap ≤ 100 bp) beyond gastrulation (Figures 1D, S1F, and S1G), with at least 97% of target genes being bound at both stages (Figure S1H). However, DNA occupancy levels of target genes did alter slightly between gastrula and early tail bud stages (Figure S1I). These results are consistent with the notion that gastrula and early tail bud embryos contain the same kind of Xbra-expressing cells, including neuromesodermal stem cells (Davis and Kirschner, 2000; Gont et al., 1993; Tzouanacou et al., 2009).

### Xbra Balances Mesodermal over Neural Cell Fates

**Figure S2:**
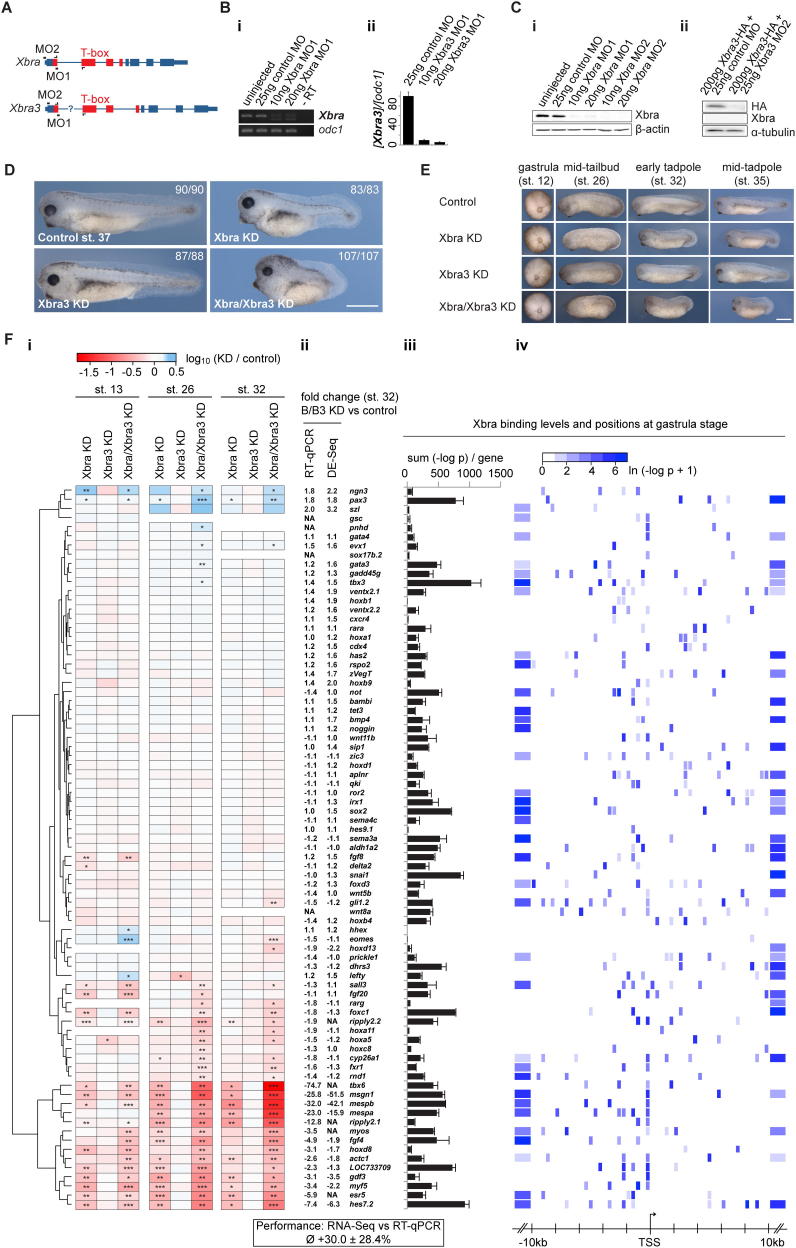
Transcriptional Misregulation of Xbra Target Genes Induced by the Single and Combined Knockdown of Xbra and Xbra3 Suggests Functional Redundancy between these Brachyury Paralogues and Reflects the Severity of Corresponding Phenotypes, Related to Figure 2 (A) Genomic architecture of *Xbra* and *Xbra3* in *X. tropicalis* including the position of the T-box within the first four exons (red). Black bars, position of splice- (MO1) and translation-blocking (MO2) morpholinos. Black ‘fish-hook’ arrows, position of PCR primers used to assess the efficiency of MO1. The length of the first intron of *Xbra3* is unknown because of a sequencing gap in the genome (JGI4.1). (B) Efficiency of blocking splicing of *Xbra* (i) and *Xbra*3 (ii) by the injection of corresponding MO1 at stage 10.5 (*Xbra*) and 12 (*Xbra3*). In contrast to 25 ng control MO, 10 or 20 ng *Xbra* MO1 caused cryptic splicing, which created multiple bands by diagnostic RT-PCR (i). *odc1*, loading control. -RT, PCR without reverse transcription. Efficiency of disrupting correct splicing of *Xbra3* was quantified by RT-qPCR taking into account melting curves to discriminate wild-type from cryptic templates. Compared to 25ng control MO, 10 or 20 ng of *Xbra3* MO1 reduced wild-type splicing to about 5%–10% (ii). Relative concentrations of *Xbra3* were normalized with *odc1*. Error bars, s.d. of biological duplicates. (C) Semiquantitative immunoblotting of endogenous Xbra protein levels at stage 11 upon injection of MO1 or MO2 at different doses (10 and 20 ng) compared to controls (uninjected and injected with 25 ng control MO) (i). Loading control, β-actin. Synthesis of 200 pg Xbra3-HA was inhibited in vivo by *Xbra3* MO2 compared to control MO (ii). ChIP-grade Xbra antibody did not recognize Xbra3-HA. Loading control, α-tubulin. (D) The embryo was consistently truncated along its antero-posterior axis, more strongly by the double Xbra/Xbra3 knock-down (KD) than the single Xbra KD. No defects were observed for Xbra3 KD embryos. By tadpole stage, Xbra/Xbra3 morphants were severely truncated to the extent that the tail was hardly elongating. Statistics given here in the top right corner of every picture were from one single experiment recorded at stage 37. Scale bar, 0.5 mm. (E) Developmental time course of embryos injected with MO1 and MO2 (5 ng each) to knock down Xbra and Xbra3 individually and simultaneously. Scale bar, 0.5 mm. (F) Clustered differential expression profile (Xbra, Xbra3 and Xbra/Xbra3 KD versus control) for some Xbra target genes: (i) Transcriptional fold changes induced by the KD of Xbra, Xbra3 or Xbra/Xbra3 were determined at stage 13 (early neurula), 26 (mid-tailbud) and 32 (early tadpole) by RT-qPCR (n = 3), logarithmised, clustered and visualized as a heat map (red, downregulated; blue, upregulated). ≥ 1.5-fold transcriptional mis-regulation: ^∗^, FDR < 10%; ^∗∗^, FDR < 1%; ^∗∗∗^, FDR < 0.1%. (ii) If available, the fold change of transcription is indicated for Xbra/Xbra3 (B/B3) KD compared to control embryos at stage 32 calculated by RT-qPCR and RNA-Seq/DESeq (DE-Seq). Floating-point numbers preceded by a minus symbol indicate a downregulation. NA indicates that differential expression data could not be obtained from the RNA-Seq experiment since the transcriptome assembly (JGI4.1) used for the alignment did not contain the sequences for *esr5*, *myos*, *ripply2.1*, *ripply2.2* and *tbx6*. Differential expression data was subsequently complemented with these missing genes. A comparison of 70 genes indicated that RNA-Seq followed by DESeq (Anders and Huber, 2010) resulted in an average 30% (s.d. +/−28.4%) greater positive fold change than RT-qPCR. (iii, iv) Xbra binding levels and positions per selected target gene at gastrula stage. (iii) Quantification of binding per target gene (sum [-log p]). Error bar, s.d. of biological duplicates. (iv) Xbra binding pattern closest to selected target genes. DNA occupancies were binned at 400-bp intervals between 10 kb up- and downstream from the corresponding TSS. Beyond this range Xbra binding events were collected into single bins. DNA occupancy was visualized as a heat map according to the natural logarithm (ln) of [-log p + 1].

Of the genes that are bound by Xbra, a few have particularly high DNA occupancies within 10 kb of the TSS (Figures 1I and S1J), and we asked whether these are regulated by this T-box TF. The *X. tropicalis* genome contains two nearly syn-expressed Brachyury paralogues, *Xbra* and *Xbra3*, and their activities were inhibited by use of splice- and translation-blocking antisense morpholino oligonucleotides (Figures S2A–S2C). In the course of these experiments, we discovered that the ChIP-grade Xbra antibody does not detect Xbra3 (Figure S2Cii), suggesting that the Xbra binding profiles do not include Xbra3 binding events. Knockdown of Xbra caused truncation of the embryonic body axis, whereas depletion of Xbra3 had little discernible effect. Depletion of both gene products caused a more severe truncation of the tail than did depletion of Xbra alone (Figures S2D and S2E).

**Figure 2:**
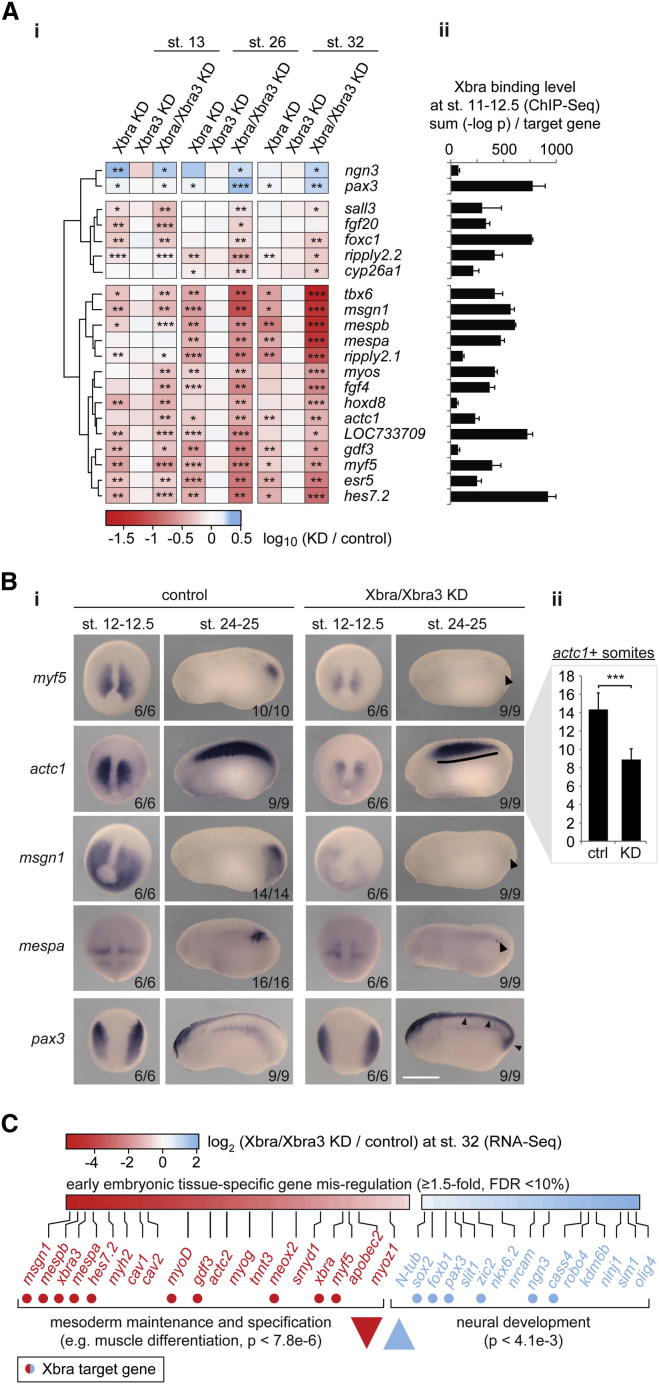
Brachyury Paralogues Xbra and Xbra3 Balance Mesodermal over Neural Fate and Prime Mesoderm for Differentiation (A) Differential expression profile (n = 3) of Xbra target genes. Transcriptional fold changes upon Xbra, Xbra3, or Xbra/Xbra3 KD determined at stage 13 (neurula), 26 (midtail bud), and 32 (early tadpole) by RT-qPCR (i), logarithmized, clustered, and visualized as heat map. Gene-associated total Xbra binding levels (ii) detected at stages 11–12.5 (gastrula) by ChIP-seq (n = 2). (B) WMISH (i) of control and Xbra/Xbra3 KD embryos for selected Xbra target genes at stages 12–12.5 (late gastrula) and 24–25 (midtail bud). Arrowheads and line indicate loss of posterior mesoderm (*myf5*, *msgn1*, *mespa*), formation of irregular, anterior somites (*actc1*), and ectopic or elevated expression within tail bud and dorsal nervous system (*pax3*). The scale bar represents 0.5 mm. (ii) Number of *actc1*+ somites formed by stage 24 and 25 in control and Xbra/Xbra3 KD embryos (n = 9). (C) Tissue-specific Gene Ontology (GO) term analysis of differentially expressed genes (≥1.5-fold; FDR < 10%) in transcriptome-wide study of control and Xbra/Xbra3 KD embryos at stage 32. Statistical significance (p) according to Mann-Whitney U test using PANTHER classification system (Mi et al., 2010). All error bars, SD of indicated biological replicates (n). ≥1.5-fold transcriptional misregulation: ^∗^, FDR < 10%; ^∗∗^, FDR < 1%; ^∗∗∗^, FDR < 0.1%. See also Figures S2, S3, and S4 and Table S2.

**Figure S3:**
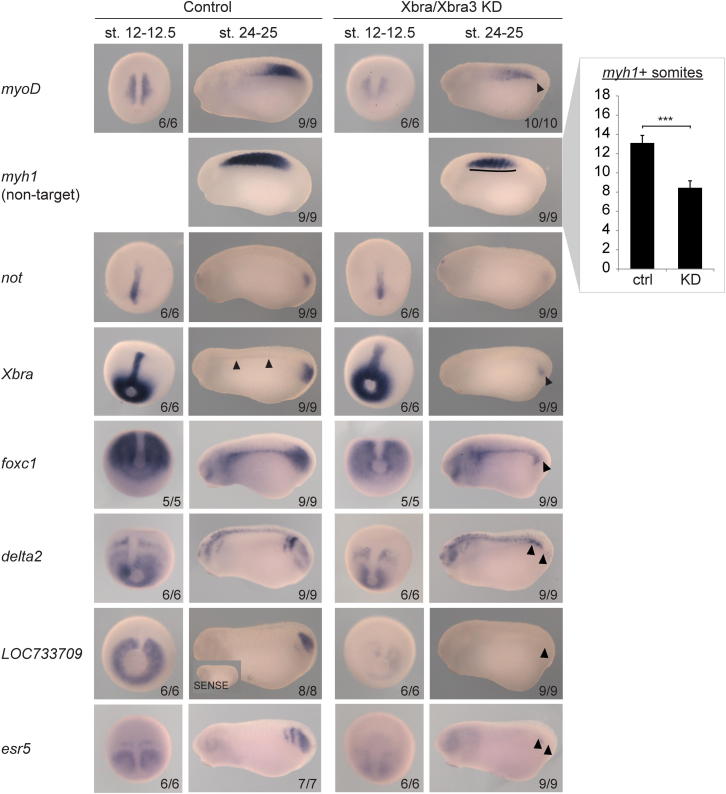
Transcriptional Misregulation of Xbra Target Genes Induced by the Combined Knockdown of Xbra and Xbra3 Affects Posterior Mesoderm Development, Related to Figure 2 WMISH on control and Xbra/Xbra3 KD embryos at late gastrula (stage 12-12.5) and mid-tailbud (24-25) stage for several Xbra target genes (*myoD*, *not*, *Xbra*, *foxc1*, *delta2*, *LOC733709* and *esr5*) and muscle differentiation marker *myh1*. Arrowheads and line indicate loss of posterior mesoderm development (*myoD*, *not*, *Xbra*, *foxc1*, *delta2*, *LOC733709* and *esr5*) and formation of irregularly shaped, anterior somites (*myh1*) upon Xbra/Xbra3 KD. Number of *myh1*+ somites (n = 9) formed by stage 24-25 plotted for control and Xbra/Xbra3 KD embryos (error bar, s.d.; two-tailed Student’s t test: ^∗∗∗^p < 0.001). The sense probe of the uncharacterized gene *LOC733709* did not produce any staining as shown on a mid-tailbud embryo. Statistics in bottom right corner indicate the number of embryos observed with the depicted WMISH pattern versus the number of embryos analyzed in total. Views: stage 12-12.5, dorso-posterior; stage 24-25, lateral, posterior end to the right. Scale bar, 0.5 mm.

Depleted embryos were transcriptionally profiled against controls by RT-quantitative PCR (qPCR) at early neurula, midtail bud, and early tadpole stages. Analysis of 78 of our putative Xbra target genes revealed that slightly less than half (37) were affected at one or more of these stages by at least 1.5-fold (FDR < 10%; Figures 2A and S2F) in either the single or double knockdown of Xbra and Xbra3. Loss of just Xbra yielded results that resembled those of the double knockdown but were less severe and in line with the weaker phenotype, suggesting that Xbra and Xbra3 act in a functionally redundant manner. Among the most significantly downregulated genes in Xbra/Xbra3 knockdown embryos were seven involved in the maintenance and specification of paraxial mesoderm and the initiation of somitogenesis at the posterior end of the embryo: *tbx6* (Chapman and Papaioannou, 1998); *msgn1* (Yoon and Wold, 2000); *mespa* (Sparrow et al., 1998); *mespb*; *ripply2.1* (Chan et al., 2006); *hes7.2*/*esr4*; and *esr5* (Jen et al., 1999). The disruption of posterior mesoderm formation (see arrowheads in Figures 2B and S3) was confirmed by whole-mount in situ hybridization (WMISH) of several of our Xbra target genes, such as *Xbra* itself and markers of muscle (*myf5*, *myoD*, and *actc1*), notochord (*not*), paraxial mesoderm (*msgn1* and *foxc1*), and nascent somites (*delta2*, *mespa*, and *esr5*). The Xbra/Xbra3 loss-of-function phenotypes, including reduced numbers of *actc1*+ or *myh1*+ somites, may derive in large part from the loss of these gene products (Figures 2B and S3). The Xbra/Xbra3-dependent target gene *LOC733709* (Figure S3), whose sequence and expression pattern is similar to that of *esr5* (Jen et al., 1999), may also be a component of the segmentation clock. We also note that the loss of Brachyury function causes significant misregulation of Xbra target genes in gastrula and early neurula embryos (Figures 2A, 2B, and S3), but this is not sufficient to completely disrupt the formation of anterior somites or to cause obvious morphological defects before the tail bud stage (Figure S2E).

Some Xbra target genes were identified as being upregulated in embryos lacking Xbra/Xbra3. These include *pax3* and *ngn3* (Figure 2A), both of which pattern the dorsal spinal cord (Bang et al., 1997; Nieber et al., 2009), and indeed, *pax3* showed increased expression in the posterior neural tube and ectopic expression in the tail bud (see arrowheads in Figure 2B), the source, at the posterior wall of the neurenteric canal, of paraxial mesoderm.

**Figure S4:**
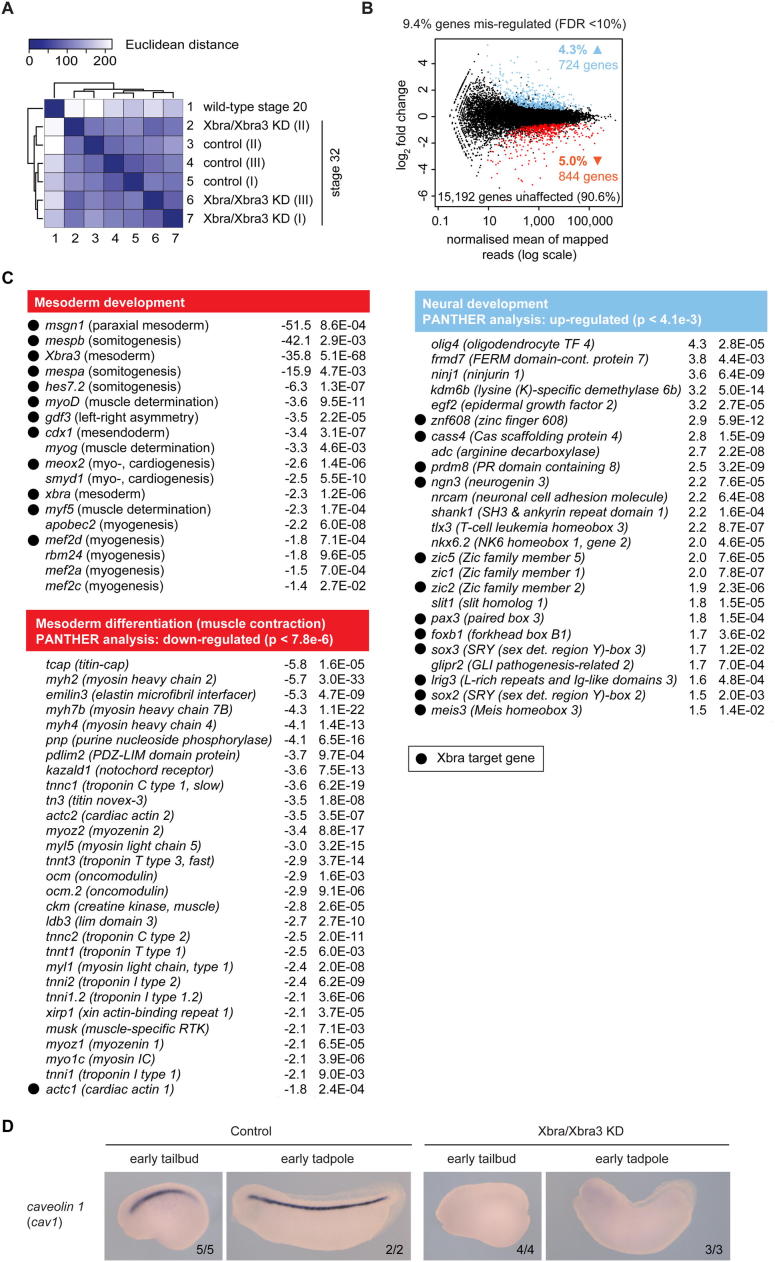
Transcriptome Analysis of Xbra/Xbra3-Depleted Early Tadpoles Confirms Cell Identity Shift from Mesoderm to Neurectoderm, Related to Figure 2 (A) Hierarchically clustered heat map of transcriptional profiles (RNA-Seq) of control and Xbra/Xbra3 KD embryos at early tadpole stage (stage 32) in biological triplicates based on Euclidean distances. Wild-type embryos (stage 20) were used as an outlier. We note considerable biological variation among biological replicates (I and III versus II) causing clustering irrespective of KD condition. (B) Plot of averaged read count between the control and KD condition versus the log_2_ fold change between Xbra/Xbra3 KD and control embryos. Under the condition of 10% FDR, 724 genes were declared as upregulated (4.3%, blue dots), 844 genes as downregulated (5.0%, red dots) and 15,192 genes as not significantly differentially expressed (90.6%). (C) Statistical significance of gene mis-regulation (≥1.5-fold, FDR < 10%) specific to mesoderm (downregulated; p < 7.8e-6) and neurectoderm (upregulated; p < 4.1e-3) was based on the Mann-Whitney U test using the PANTHER classification system (Mi et al., 2007). Early embryonic tissue-specific data sets were manually curated. Tables list mis-regulated genes (including fold changes and p-values adjusted with the Benjamini-Hochberg procedure) mainly associated with the biological process of mesoderm development (gene abbreviations are followed by the gene-associated mesodermal process in parentheses), muscle contraction (PANTHER GO term) and neural development (PANTHER GO term). A minus preceding the floating-point number of the fold change indicates a downregulation. Genes marked with a dot were associated with Xbra binding. (D) WMISH for *caveolin-1 (cav1)* at early tailbud and early tadpole stage illustrates the loss of notochord development upon Xbra/Xbra3 KD. Statistics in bottom right corner indicates the number of embryos observed with the depicted WMISH pattern versus the number of embryos analyzed in total.

To substantiate Brachyury-dependent down- and upregulation, respectively, of mesoderm-specific and neurogenic genes, control and Xbra/Xbra3 knockdown embryos were subjected to transcriptome-wide profiling (RNA-seq) at the early tadpole stage (Figure S4A; Table S2), when the knockdown phenotype was most pronounced and RT-qPCR suggested that transcriptional misregulation might be most dramatic. Loss of Xbra/Xbra3 caused misregulation of 1,568 (FDR < 10%) out of 16,760 genes (9.4%), with about half downregulated and half upregulated (Figure S4B). Among the downregulated genes were *Xbra3* (35.8-fold), the notochord markers *cav1* (5.1-fold) and *cav2* (4.5-fold), and an overrepresented group (Mann-Whitney U test; p < 7.8 × 10^−6^) of genes expressed in muscle, including *myh2* (5.9-fold) and *tnnc1* (3.6-fold). Upregulated genes enriched for neural differentiation (p < 4.1 × 10^−3^) included Xbra targets, such as *sox2* (1.5-fold), *foxb1* (1.7-fold), *pax3* (1.8-fold), *zic2* (1.9-fold), and *ngn3* (2.2-fold; Figures 2C and S4C). These results confirm that loss of mesodermal identity, including muscle (Figure 2C) and notochord (Figure S4D), is accompanied by elevated expression of several neural genes, some of which are Xbra targets.

### DNA Occupancy Pattern of Xbra Correlates with Gene Activation

Loss of Brachyury caused downregulation of some target genes and upregulation of others. We asked whether the level and position of binding might discriminate between these genes (≥1.5-fold; FDR < 10%) and unaffected target genes.

**Figure 3:**
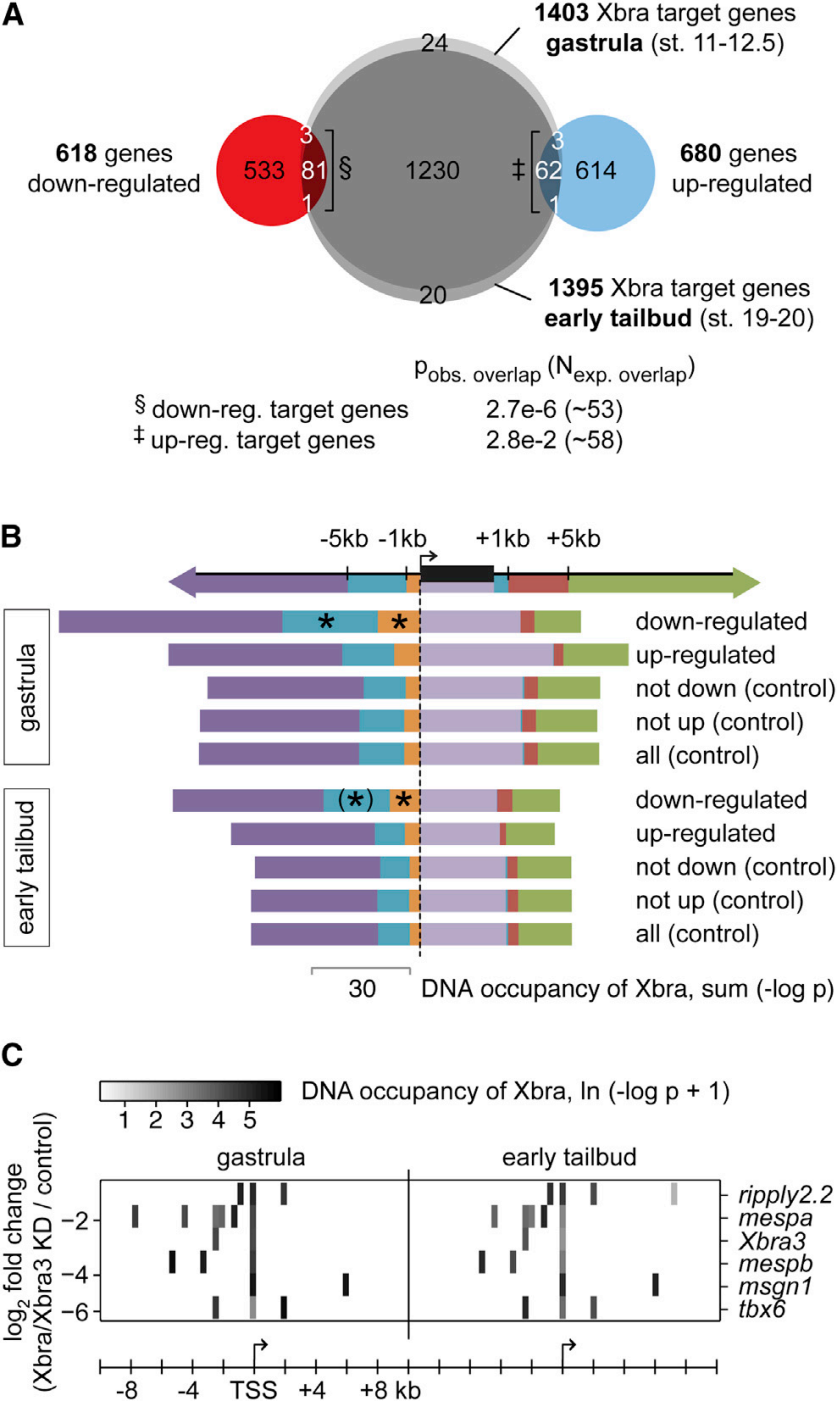
Strongly Activated Target Genes Show Preferential Xbra Binding to Promoter-Proximal and Intermediate Upstream Regions (A) Venn diagram of genes targeted by Xbra at gastrula and/or early tail bud stages (sum [−log p_gastrula_] or sum [−log p_tailbud_] ≥ 25) and genes misregulated at stage 32 (≥1.5-fold; FDR < 10%) following Xbra/Xbra3 KD. Fisher’s exact test indicates probability of observed overlap (p_obs. overlap_) and expected number of overlap (N_exp. overlap_) based on random draws of gene sets from 16,760 genes (for which differential expression was calculated in Table S2). (B) Xbra binding at gastrula and early tail bud stage across down- or upregulated target genes (≥1.5-fold; FDR < 10%) compared with control sets of target genes. The asterisk indicates significantly (p < 0.05) enriched binding compared to controls according to a one-tailed Mann-Whitney U test (Extended Experimental Procedures). Brackets indicate loss of statistical significance (p ∼ 0.2) when zero DNA occupancies were excluded. See also Table S3. (C) Heat map representation of Xbra binding near strongly Xbra/Xbra3-dependent target genes at gastrula and early tail bud stages.

To this end, gastrula and early tail bud Xbra binding profiles were compared with Xbra/Xbra3 loss-of-function analyses at the early tadpole stage (Figure 3A). Downregulated and upregulated genes both overlapped to a small but significant extent with Xbra binding profiles (Fisher’s exact test; p < 0.05), with more downregulated target genes than upregulated. Similarly, compared to other target gene sets, downregulated genes showed a higher, statistically significant (p < 0.05) binding level than upregulated genes at both proximal (<1 kb) and intermediate distances (1–5 kb) from their TSSs (Figures 3B; Table S3). This binding pattern was particularly prominent at target genes, such as *ripply2.2*, *mespa/b*, *Xbra3*, and *tbx6*, whose transcription was strongly activated by Xbra/Xbra3 (Figure 3C). This group of genes also showed a slight enrichment for motif v1 when compared to all other gene sets, suggesting that affinity may play a role in regulating transcription (data not shown).

### T-box TF Family Members Bind and Regulate Overlapping Genes

**Figure 4:**
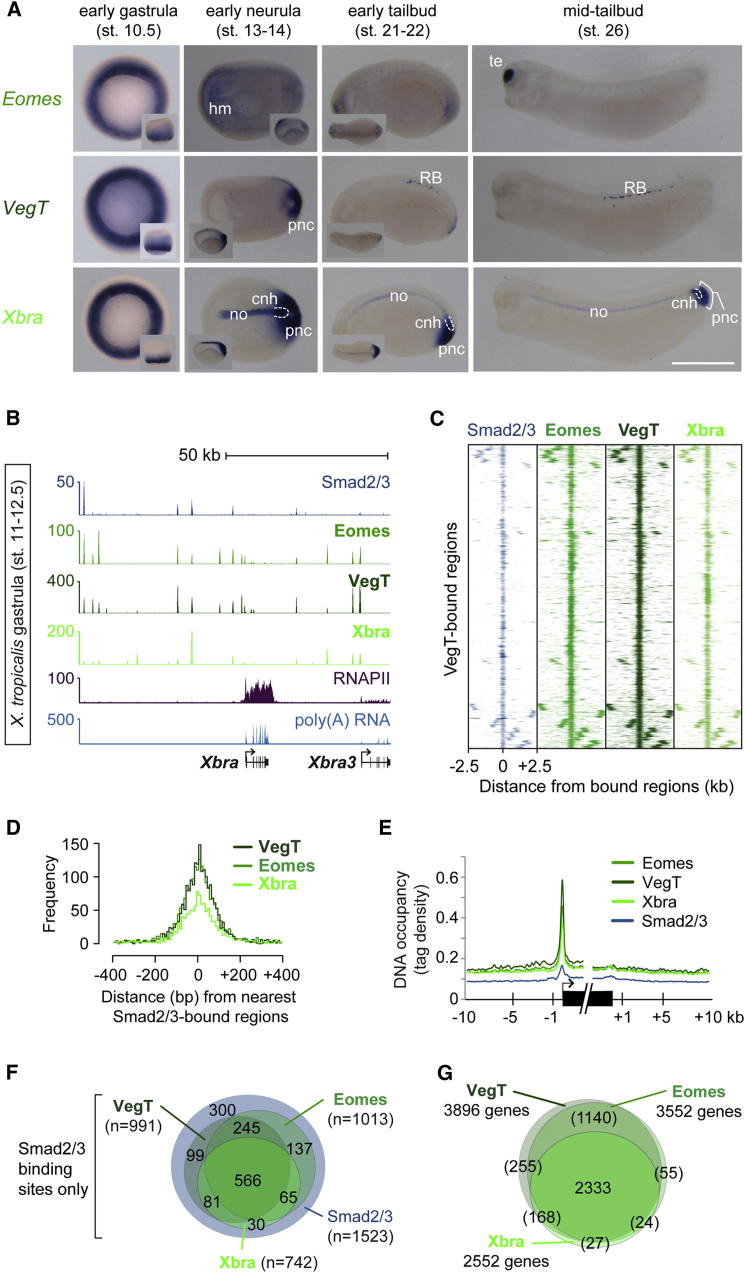
Eomes, VegT, and Xbra Occupy the Same Genomic Recognition Sites and TGF-β Responsive Regulatory Elements (A) Developmental series of WMISHs for *Eomes*, *VegT*, and *Xbra*. cnh, chordoneural hinge; hm, head (prechordal) mesoderm; no, notochord; pnc, posterior wall of neurenteric canal; RB: Rohon-Beard cells; te, telencephalon. The scale bar represents 0.5 mm. (B) Gastrula-staged snapshot of Eomes, VegT, Xbra, and Smad2/Smad3 (Yoon et al., 2011) binding near *Xbra* and *Xbra3*. (C) Heat maps represent DNA occupancies of Smad2/Smad3, Eomes, VegT, and Xbra relative to all VegT-bound regions during gastrulation. (D) Histograms based on pairwise calculations of shortest distances between T-box TF and Smad2/Smad3 binding positions (FDR ≤ 1%) during gastrulation. (E) Metagene model shows DNA occupancy level of Eomes, VegT, Xbra, and Smad2/Smad3 relative to the start and end of nearest target genes. (F) Venn diagrams for Smad2/Smad3 binding positions overlapping (distance ≤ 100 bp) with the binding position of Eomes, VegT, and/or Xbra (FDR ≤ 1%). (G) Venn diagrams for Eomes, VegT, and Xbra target genes. The stringency was relaxed up to p ≤ 0.1 unless DNA occupancy was found equivalent to p ≤ 10^−25^ when comparing binding between two T-box TFs. DNA occupancies (p ≤ 10^−25^ + 10^−25^ < p ≤ 0.1 = total) detected as follows: Eomes (2407 + 1145 = 3552); VegT (3628 + 268 = 3896); Xbra (1379 + 1173 = 2552). The extent of overlap between genes targeted by different T-box TFs might be greater than indicated in brackets, because peaks (p ≤ 0.1) sometimes failed to be detected. See also Figure S5 and Table S1.

**Figure S5:**
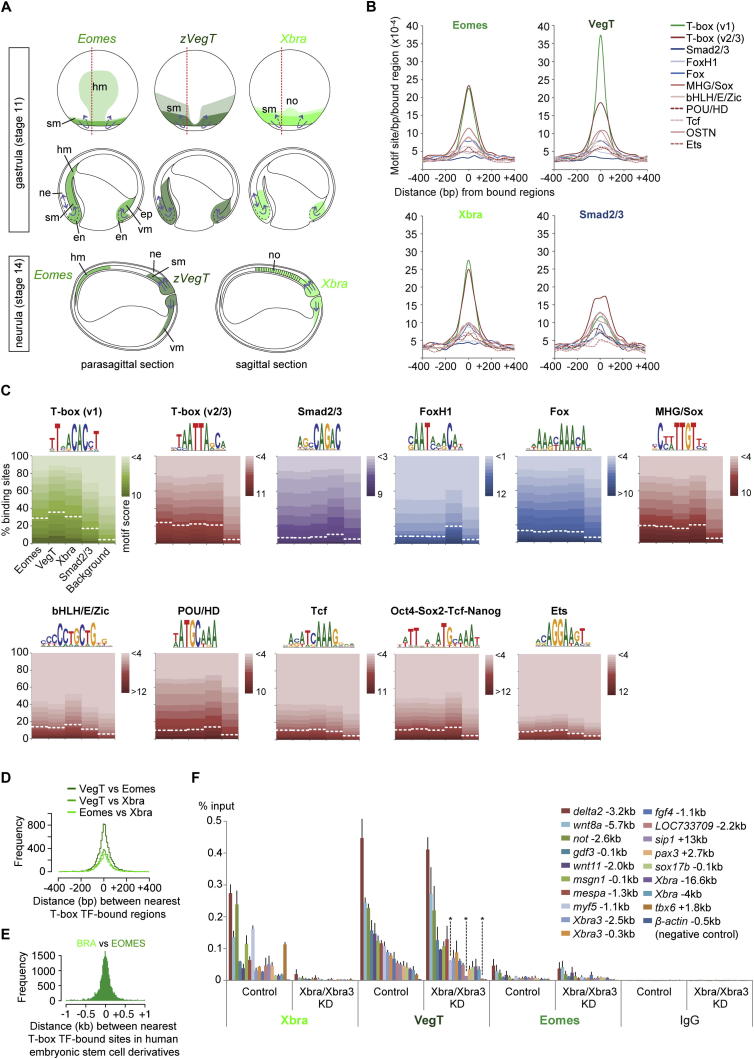
Expression Patterns and Binding Characteristics of Eomes, VegT, and Xbra in *Xenopus* Embryos during Gastrulation, Related to Figure 4 (A) Expression pattern scheme of *Eomes*, *VegT* and *Xbra* at gastrula (dorsal view and parasagittal section as indicated with ventral side to the right) and early neurula (sagittal and parasagittal section with posterior end to the right) based on our own WMISH and WMIHC (not shown) and published expression data (Fukuda et al., 2010; Horb and Thomsen, 1997; Ryan et al., 1996; Smith et al., 1991; Stennard et al., 1996, 1999; Zhang and King, 1996). At early gastrula stage, *Eomes* and *VegT transcripts* are also found in vegetal endoderm (omitted in figure). Blue arrows, morphogenetic movements (Stern, 2004). Abbreviations: en, endoderm; ep, epidermis; hm, head mesoderm; ne, neurectoderm; no, notochord; sm, somitic mesoderm; vm, ventral mesoderm. (B) Density maps of enriched motifs found in genomic regions bound by Eomes, VegT, Xbra and Smad2/3 in vivo show that their bindings correlate with highest enrichment for the T-box TF motif. (C) Motif enrichments for several DNA-binding factors were calculated from genome-wide binding maps for Eomes, VegT, Xbra (this study) and Smad2/3 (Yoon et al., 2011) using Homer Perl scripts (Heinz et al., 2010) and normalized to 5% background (see dashed line in diagrams for each motif). The underlying motif score was used to create the density maps (B). We note that the consensus motif of FoxH1 known to interact with Smad2/3 (Chen et al., 1997; Watanabe and Whitman, 1999; Zhou et al., 1998) is as enriched at Smad2/3 binding sites as T-box or MHG/Sox motifs. The same MHG/Sox motif is also found at T-box TF binding sites. (D) Histograms based on pairwise (Xbra versus Eomes, Xbra versus VegT and Eomes versus VegT) calculations of distances between the nearest peaks (FDR ≤ 1%) of two different T-box TFs in frog gastrula embryos. (E) Histograms based on pairwise calculations of shortest distances between BRACHYURY and EOMES binding positions in human mesoderm and definitive endoderm (Teo et al., 2011) derived from embryonic stem cells in vitro. (F) ChIP-qPCR for Xbra, VegT and Eomes on control and Xbra/Xbra3-depleted late gastrula embryos (stage 12). DNA occupancy plotted as percentage of DNA input. Error bars, s.d. of biological duplicates. Two-tailed Student’s t test indicates significant reduction (^∗^) of VegT recruitment to genomic regions 1.1kb upstream of *myf5* (p = 0.11), 1.8kb upstream of *tbx6* (p = 0.11) and 13kb downstream of *sip1* (p = 0.07) upon Xbra/Xbra3 KD. All other VegT and Eomes binding events seem not to be or non-significantly affected by the KD of Xbra/Xbra3 at stage 12.

Embryos lacking Xbra/Xbra3 gastrulate normally (Figure S2E) and form mesodermal structures anterior to somites 8–12 (e.g., *actc+* somites in Figure 2B). Other T-box TFs may complement *Xbra*/*Xbra3* to allow the formation of these anterior tissues. To test this, we extended our study to include *Eomes* and zygotic *VegT*, whose expression patterns around the blastopore and the posterior wall of the neurenteric canal resemble that of *Xbra* during gastrulation and neurulation, with the exception of the chordoneural hinge and notochord (Figures 4A and S5A).

DNA occupancies of Eomes and VegT were determined by ChIP-seq. Despite their different loss-of-function phenotypes and in vitro affinities for DNA sequences (Conlon et al., 2001; Fukuda et al., 2010), Eomes and VegT were recruited to the same genomic sites as Xbra during gastrulation (Figures 4B, 4C, and S5D) such that most, if not all, target genes were occupied by at least two of these T-box TFs (Figure 4G), suggesting that all three recognize the same binding motifs in vivo (Figures S5B and S5C). A comparison of Brachyury and Eomes (Teo et al., 2011) binding in mesoderm and definitive endoderm derived from human embryonic stem cells reached a similar conclusion (Figure S5E). However, we have no evidence for competition between T-box TFs for individual T-box recognition sites, because Eomes and VegT binding did not increase at Xbra/Xbra3-depleted sites during gastrulation (Figure S5F). This might explain why embryos could not fully compensate for gene misregulation caused by the loss of Brachyury (Figures 2A, 2B, and S2F), and it suggests that there may be only limited overlap of T-box protein expression in single cells or poor accessibility for other T-box TFs at Xbra/Xbra3-depleted sites. Interestingly, the loss of Xbra/Xbra3 caused a significant reduction of DNA occupancy of VegT at some sites, suggesting that some VegT binding is Xbra/Xbra3-dependent. Despite the great similarity of T-box TF binding profiles, the three T-box TFs differed in their DNA occupancies of particular sites (see peaks in Figures 4B and S5F).

Further analysis revealed that over 80% of the binding positions of nuclear Smad2/Smad3 (Yoon et al., 2011), which mediates transforming growth factor β (TGF-β) signaling and targets responsive *cis*-regulatory elements on the genome, overlap with those of the T-box TFs during gastrulation (Figures 4B–4F). This supports the idea that Smads and T-box TFs may act together (Teo et al., 2011) to regulate target gene expression underlying primary cell fate decisions at these stages. There is little enrichment for Smad2/Smad3 motifs at bound sites (Figures S5B and S5C), suggesting that T-box proteins and perhaps members of other TF families contribute to the recruitment of Smad2/Smad3 to their binding sites (Mullen et al., 2011).

**Figure 5:**
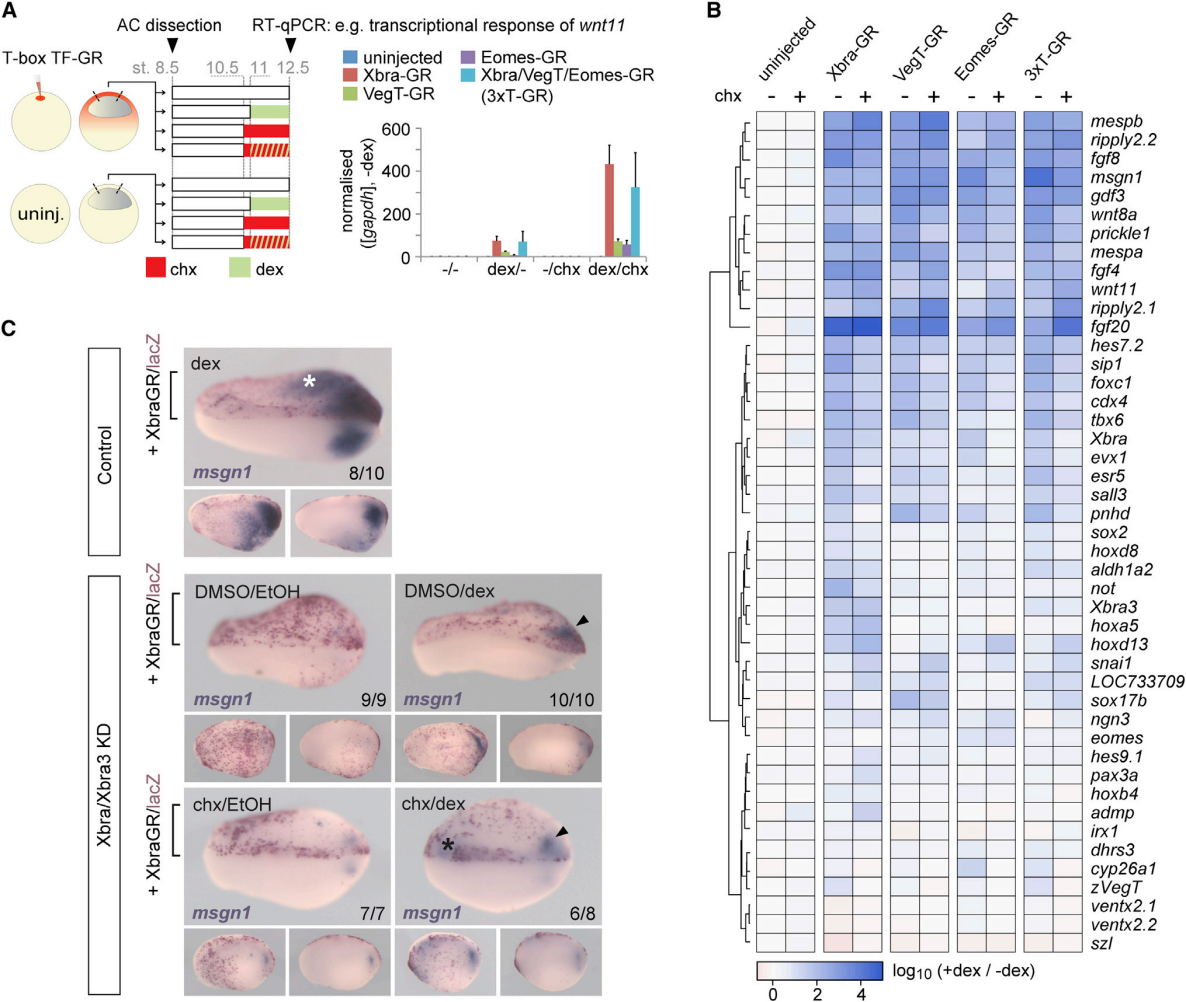
Eomes, VegT, and Xbra Can Activate Directly the Same T-box TF-dependent Target Genes (A) Animal cap (AC) assay to detect direct regulation (i.e., in the presence of chx) of gene transcription by RT-qPCR using dex-inducible fusion constructs (Xbra-GR, VegT-GR, Eomes-GR) individually or in combination (3xT-GR). Data normalized to *gapdh* and the uninduced sample (−dex). The error bars represent SD of biological duplicates. (B) Heat map representation of clustered transcriptional response ratios (+dex/−dex) of T-box TF target genes to the activity of T-box TFs with (−chx) or without (+chx) de novo protein synthesis. (C) Protein synthesis-independent rescue of *msgn1* transcription in the tail bud (arrowheads) of Xbra/Xbra3-depleted embryos (stages 22–23) by activated Xbra-GR, whose RNA was unilaterally injected together with *lacZ* lineage tracer RNA.

The genome-wide binding characteristics of Eomes, VegT, and Xbra suggest that they regulate the same genes. To test this possibility directly, we used an animal cap assay. Gene activation by hormone-inducible versions (glucocorticoid receptor [GR]) of Eomes, VegT, and Xbra was analyzed in the presence and absence of the protein synthesis inhibitor cycloheximide (chx) to ask whether induction was direct (Figure 5A). Forty-five target genes were analyzed (Figures 5B). Most target genes that were downregulated in embryos lacking Brachyury (including *mespb*, *ripply2.2*, *fgf8*, *msgn1*, *gdf3*, *mespa*, *fgf4*, *ripply2.1*, *fgf20*, *hes7.2*, *foxc1*, and *esr5*) were activated directly by all three T-box TFs. Target genes that were upregulated in such embryos (such as *szl, pax3*, and *ngn3*) were not activated or were only weakly so. There were some differences in the inducing activities of the T-box TFs, however. For example, *not* and *Xbra3* were significantly induced only by Xbra, and indeed, Eomes and VegT repressed their expression (Figure 5B). Similarly, *tbx6* and *LOC733709* were activated by Xbra and VegT but not Eomes, and the endodermal marker *sox17b* was preferentially induced by VegT. These differences may arise through the differential recruitment of transcriptional cofactors by the different T-box TFs. Analogous experiments within the whole embryo revealed that Xbra-GR can partially restore *msgn1* expression within the tail bud of Xbra/Xbra3-depleted embryos both in the presence and in the absence of de novo protein synthesis (see arrowhead in Figure 5C). Exogenous Xbra activity was also able to drive ectopic *msgn1* transcription in mesodermal and, less frequently, nonmesodermal tissues (see asterisks in Figure 5C).

### T-box TFs Recruit RNA Polymerase II to Define Neuromesodermal Bipotency

**Figure 6:**
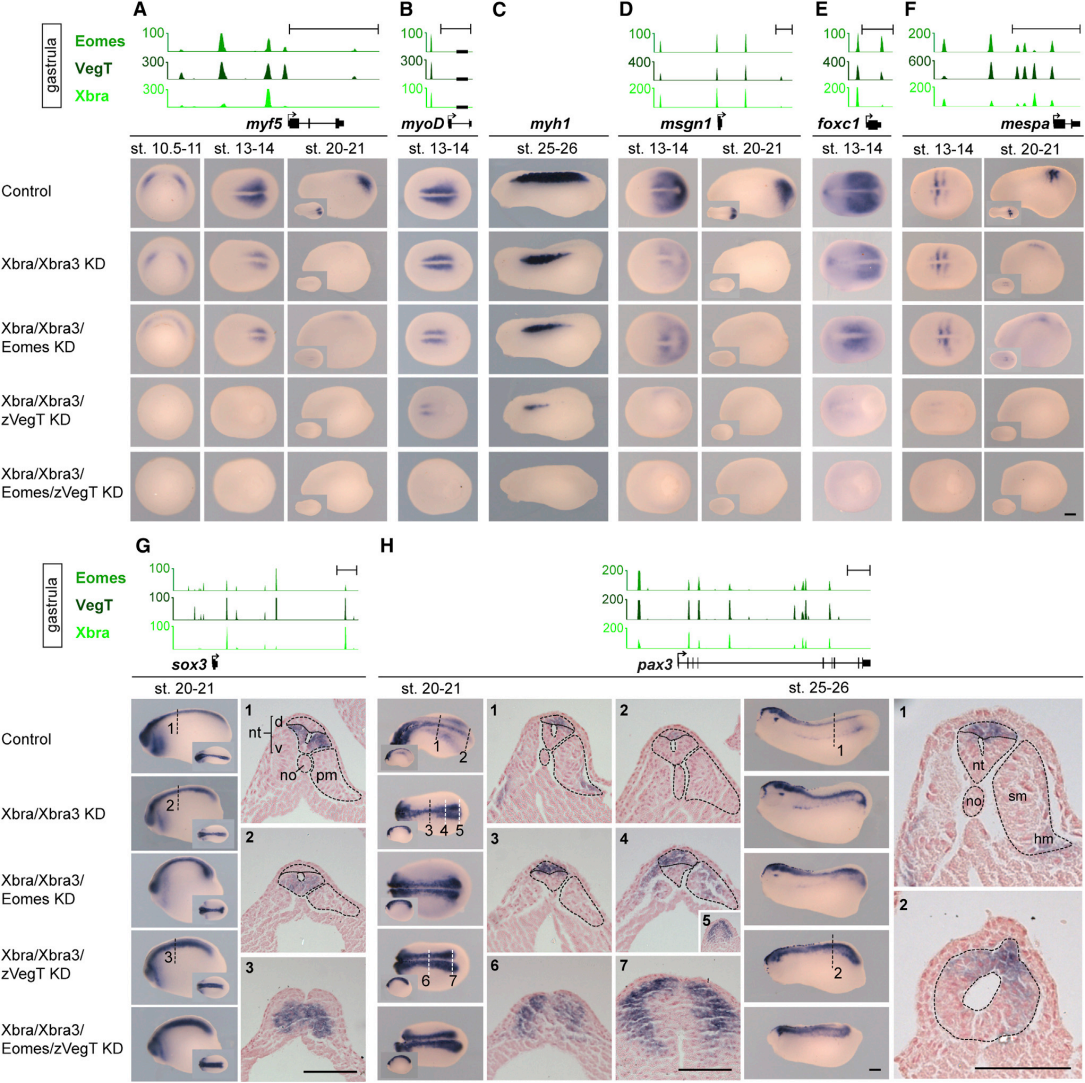
Combined Loss of Eomes, VegT, and Xbra Reveals Their Collaboration to Determine Neuromesodermal Bipotency and Promote Mesodermal Differentiation during Axial Elongation Gastrula-staged snapshots of Eomes, VegT, and Xbra binding near mesoderm-specific genes *myf5*, *myoD*, *msgn1*, *foxc1*, *mespa* (A, B, and D–F), and neurogenic genes *sox3* and *pax3* (G and H). WMISH on control and indicated KD embryos for target genes (A, B, and D–H) and muscle-specific differentiation marker *myosin heavy chain 1*, *myh1* (C). Cross-sections at positions of *sox3* and *pax3* WMISH as indicated. no, notochord; nt, neural tube (d, dorsal; v, ventral); hm, hypaxial muscle; pm, paraxial mesoderm; sm, skeletal muscle. The scale bar represents 0.2 mm. See also Figures S6 and S7.

**Figure S6:**
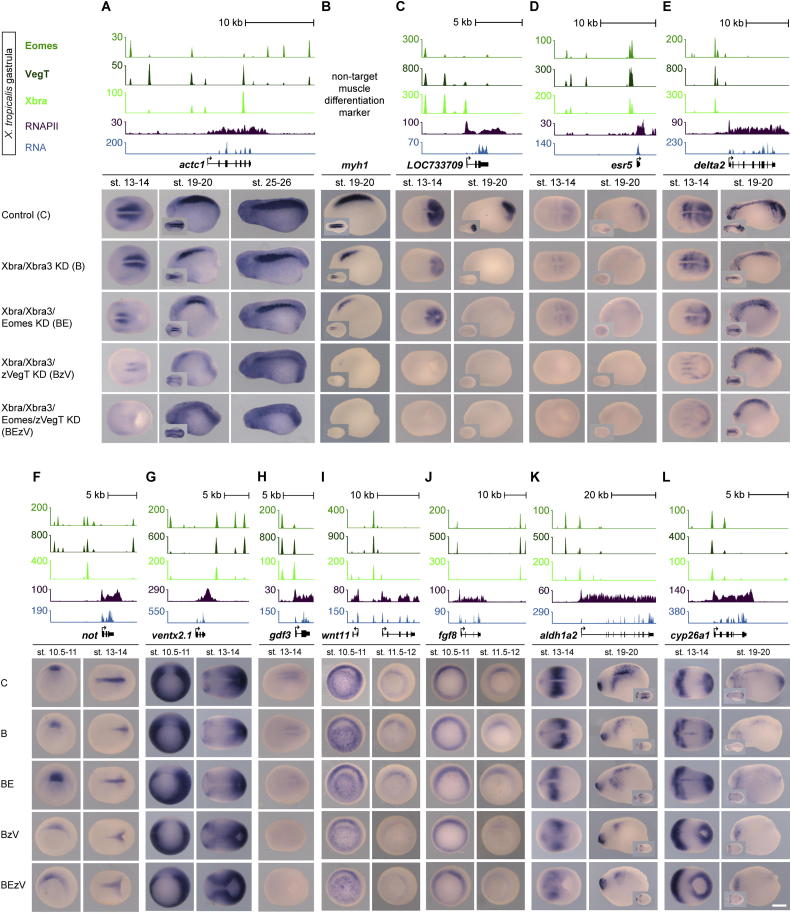
Combined Loss of T-box TFs Abrogates Neuromesodermal Bipotency, Paraxial Mesoderm Development, and other Mesoderm-Associated Developments, Related to Figure 6 Gastrula-staged snapshots of Eomes, VegT and Xbra binding nearby *actc1* (A), *LOC733709* (C), *esr5* (D), *delta2* (E), *not* (F), *ventx2.1* (G), *gdf3* (H), *wnt11* (I), *fgf8* (J), *aldh1a2* (K) and *cyp26a1* (L). RNAPII and poly(A) RNA transcript profiles are taken from Akkers et al. (2009). WMISH on control, Xbra/Xbra3, Xbra/Xbra3/Eomes, Xbra/Xbra3/zVegT and Xbra/Xbra3/Eomes/zVegT KD embryos for target genes (A),(C)-(L) and the muscle-specific differentiation marker *myosin heavy chain 1* (*myh1*) (B) at distinct developmental stages. Early (stage 10.5-11) and late (stage 11.5-12) gastrula, vegetal view; early neurula (stage 13-14), dorsal view; early tailbud (stage 19-20), lateral view (small image, dorsal view); mid-tailbud (stage 25-26), lateral view; embryos from stage 13-20, posterior to the right. Scale bar, 0.5 mm.

**Figure S7:**
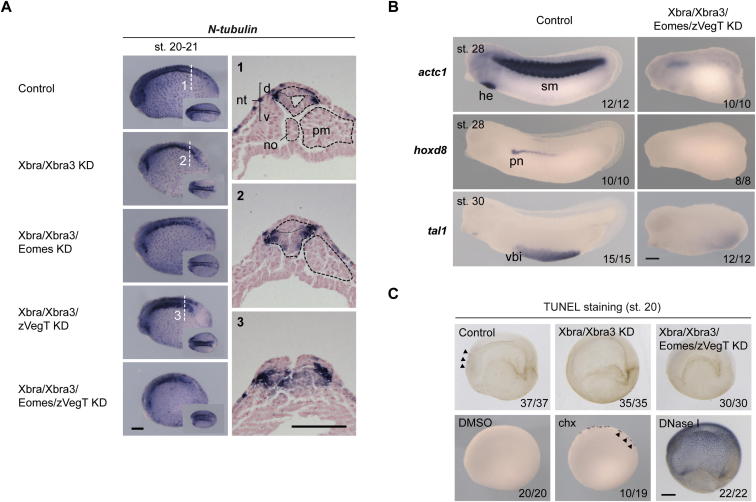
Combined Loss of T-box TFs Causes Embryos to Produce Excess Neural Tissue at the Expense of Axial and Paraxial Mesoderm and in the Absence of Apoptosis, Related to Figure 6 (A) WMISH on control, Xbra/Xbra3, Xbra/Xbra3/Eomes, Xbra/Xbra3/zVegT and Xbra/Xbra3/Eomes/zVegT KD embryos for neural differentiation marker *N-tubulin* at early tailbud stage (stage 20-21, lateral view). 1, 2 and 3 mark the position of cross-sections through control, Xbra/Xbra3 KD and Xbra/Xbra3/zVegT KD embryos. Abbreviations: no, notochord; nt, neural tube (d, dorsal; v, ventral); pm, paraxial mesoderm. (B) WMISH for *actc1*, *hoxd8* and *tal1* at late tailbud stage illustrates the loss of mesodermal derivatives such as skeletal muscle (sm), heart (he), pronephros (pn) and ventral blood island (vbi) upon Xbra/Xbra3/Eomes/zVegT KD. Statistics in bottom right corner indicates the number of embryos observed with the depicted WMISH pattern versus the number of embryos analyzed in total. (C) TUNEL staining on control, Xbra/Xbra3 and Xbra/Xbra3/Eomes/zVegT KD embryos (cleared with Murray’s clear) at early tailbud stage (stage 20). Positive controls, embryos treated for 4 hr in 35 μM cycloheximide (chx) and fixed embryos incubated with DNase I. Arrowheads mark apoptotic cells in the brain region (where apoptosis can occasionally be observed in embryos even under normal conditions) and the posterior nervous system induced by cycloheximide. Scale bar, 0.2 mm.

The similar binding profiles and regulatory capacities of Xbra, Eomes, and VegT encouraged us to explore potential collaboration between these T-box TFs in paraxial mesoderm formation. This was achieved by simultaneous knockdown of the gene products by previously verified antisense morpholino oligonucleotides (Fukuda et al., 2010). Loss-of-function of *Eomes*, zygotic *VegT*, or both, in addition to *Xbra/Xbra3*, caused a downregulation of mesodermal target genes in the trunk that exceeded that observed following knockdown of Xbra/Xbra3 alone (Figures 6A–6F and S6A–S6E). VegT, whose zygotic expression persists at the caudal end of the embryo until the early tail bud stage (Figure 4A), contributes more than Eomes to the ongoing process of presomitic mesoderm specification (*msgn1* and *foxc1*), somitogenesis (*delta2*, *mespa, LOC733709*, and *esr5*), determination (*myf5* and *myoD*), and differentiation (*actc1*) of skeletal muscle (Figures 6A, 6B, 6D–6F, S6A, and S6C–S6E). The loss of all T-box TFs impaired gastrulation and abolished the formation of mesoderm and of its derivatives, such as muscle, heart, blood, and pronephros (Figure S7B).

**Figure 7:**
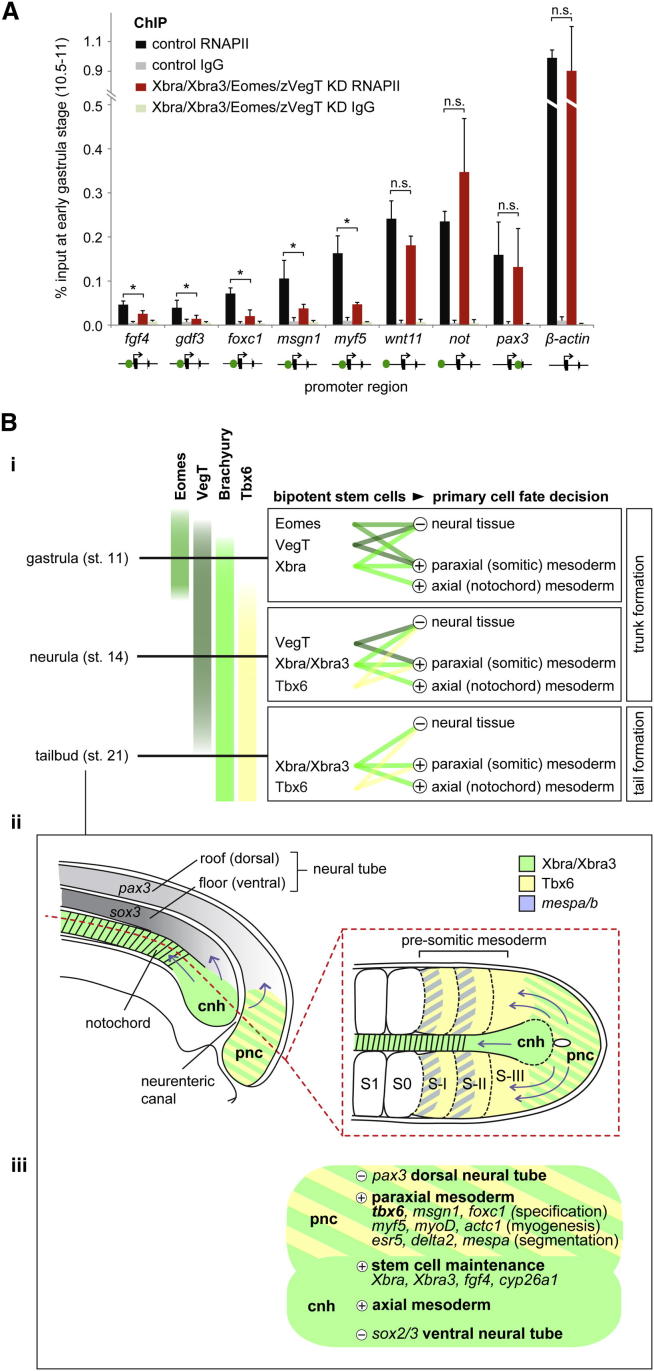
T-box TF-Dependent Recruitment of RNAPII and Model for the Way in which Stage-Dependent Combinations of T-box TFs Define and Instruct Bipotential Stem Cells to Be Recruited to Neural and Mesodermal Tissues and Prime Mesoderm for Differentiation (A) RNAPII deposition at promoters of mesodermal (*fgf4*, *gdf3*, *foxc1*, *msgn1*, *myf5*, *wnt11*, *not*), neural (*pax3*), and house-keeping (*β-actin*) genes in control and T-box TF-depleted embryos at early gastrula stage (10.5–11) determined by ChIP-qPCR. Proximal or distal (upstream or intronic) binding of T-box TFs to indicated gene promoter is symbolized with green dot. The error bars represent SD of biological triplicates. Two-tailed Student’s t test: ^∗^p < 0.1; n.s., not significant (p ≥ 0.1). IgG, immunoglobulin G. (B) Model: (i) The different spatial and temporal patterns of T-box TFs cause neuromesodermal stem cells to be defined by Eomes, VegT, and Xbra during gastrulation; VegT, Xbra, Xbra3, and Tbx6 during neurulation; and Xbra, Xbra3, and Tbx6 during tail bud stages. These combinations of T-box TFs also ensure that the correct ratio of mesodermal over neural tissue is formed during trunk and tail formation by activating mesoderm-specifying genes and repressing neurogenic genes. The development of axial (notochord) mesoderm depends mainly on Xbra/Xbra3, due to their exclusive expression among these T-box TFs in the chordoneural hinge and developing notochord. Other mesodermal derivatives, such as heart, may similarly depend on combinations of T-box TFs (omitted from model). (ii) Schematic diagram of a sagittal section and a horizontal section (red dashed line) through the posterior region of an early tail bud embryo illustrating the expression of *Xbra*/*Xbra3*, *Tbx6*, and *mespa/b* and the recruitment of mesodermal and neural cells (blue arrows) from the stem niche (chordoneural hinge and posterior wall of the neurenteric canal). Most cells of the chordoneural hinge give rise to the notochord and the ventrolateral horns of the neural tube, whereas cells in the posterior wall of the neurenteric canal contribute to paraxial (presomitic) mesoderm and the dorsal roof of the neural tube. cnh, chordoneural hinge; pnc, posterior wall of neurenteric canal; S1, first somite; S0, newly forming somite; S-I/II/III, presomitic mesoderm. (iii) Genetic regulatory inputs of T-box TFs in early tail bud embryos with several functional nodes being active in different domains (cnh, pnc) of the tail bud: stem cell maintenance; specification of somitic mesoderm; myogenic differentiation; patterning of presomitic mesoderm; notochord formation; and protection from neuralization.

Among target genes whose expression was significantly downregulated in such embryos were some involved in left-right asymmetry, such as *gdf3* (Hanafusa et al., 2000), and some in retinoic acid signaling, such as *aldh1a2* and *cyp26a1* (Deimling and Drysdale, 2009; Martin and Kimelman, 2010; Figures S6H, S6K, and S6L). Some target genes involved in morphogenesis and in the maintenance of mesodermal tissue, such as *wnt11* (Tada and Smith, 2000) and *fgf8* (Schulte-Merker and Smith, 1995), showed slight downregulation toward the end of gastrulation, whereas *not* and *ventx2.1* remained robustly expressed during gastrulation, even in the absence of all T-box TFs, suggesting that other factors are required for their regulation (Figures S6F, S6G, S6I, and S6J). In line with statistical tests outlined in Figure 3B, visual inspection of all three T-box TF binding profiles indicated that promoter-proximal binding might determine whether target genes are strongly induced by T-box TFs in vivo (e.g., Figures 6A, 6D, 6E, and S6H) or not (e.g., Figures 6H, S6F, and S6I). Indeed, ChIP analysis of T-box TF-depleted early gastrula embryos (stages 10.5–11) confirmed that the recruitment of RNA polymerase II (RNAPII) depends on T-box TFs only at mesodermal target genes, which feature promoter-proximal binding of T-box TFs, such as *fgf4*, *gdf3*, *foxc1*, *msgn1*, and *myf5* (Figure 7A). In contrast, mesodermal or neural target genes without promoter-proximal occupancy of T-box TFs, such as *wnt11*, *not*, and *pax3*, did not show any significant reductions in RNAPII deposition upon T-box TF knockdown.

The upregulation of neurogenic target genes in embryos lacking Xbra/Xbra3 was enhanced by the loss of Eomes and zygotic VegT (Figures 6G and 6H). Cross-sections through the tail buds of such embryos demonstrated the transition of mesodermal to neural identity in the chordoneural hinge (*sox3*) and posterior wall of the neurenteric canal (*pax3*; Figures 6G, 6H, and 7B), whereas the emergence of supernumerary *N-tubulin*-positive primary neurons (Figure S7A) provided further evidence of increased neural differentiation. Thus, embryos lacking all T-box TFs in the tail bud formed an oversized neural tube in the absence of any axial or paraxial mesoderm (Figure 6H). This neuromesodermal conversion occurred without significant apoptosis in the tail bud, as shown by terminal deoxynucleotidyl transferase deoxyuridine triphosphate nick end labeling (TUNEL) assays of whole-mount embryos lacking T-box TFs (Figure S7C).

## Discussion

Our results provide several lines of evidence that the T-box TFs Eomes, VegT, and Xbra/Xbra3 (and probably Tbx6) together constitute genetic regulatory inputs that define bipotential stem cells at the caudal end of the frog embryo and instruct their continuous and correct recruitment to neural and mesodermal tissues (Figure 7B). First, the combinatorial loss of T-box TFs causes embryos to generate more neural cells at the expense of mesoderm without significant induction of programmed cell death. The residual generation of somitic mesoderm (the first 8–12 somites) observed in vertebrates lacking Brachyury (Chesley, 1935; Martin and Kimelman, 2008; this study) occurs through the early action of remaining T-box TFs such as VegT and Eomes (Figure 7Bi). The loss of a T-box TF collective, including Eomes, zygotic VegT, and Brachyury (which strongly activates the expression of Tbx6) abolishes neuromesodermal bipotency and causes caudal cells to form neural tissue with complete loss of mesoderm. Second, gain-of-function experiments confirm that Eomes, VegT, and Xbra can directly activate most T-box TF-dependent target genes regulating neuromesodermal stem cell maintenance and posterior mesoderm development. And third, genome-wide binding profiles reveal that Eomes, VegT, and Xbra are recruited to the same mono- and dimeric recognition sites during gastrulation and that Xbra maintains its binding profile at least throughout the early phases of axial elongation. A nuclear concentration of Xbra quantified as ∼2.9 μM at the midgastrula stage suggests that there is a high occupancy rate of accessible recognition sites, especially at the most stringently conserved motif, whose dissociation constant is ∼14 nM.

The observation that T-box TFs are recruited to the same sites bears on the interpretation of experiments involving dominant-interfering TF constructs. For example, an Xbra-En^R^ construct may well inhibit the function of Eomes, VegT, and Tbx6, as well as that of Xbra. Collaborations between TFs may also be encountered in other TF families, including the Sox proteins, several of which define the nervous system (Bergsland et al., 2011).

Despite the thousands of T-box TF binding sites detected in our genome-wide study, only a minority of these binding events seem to cause biologically significant changes in transcription. We show that genes that are strongly activated by T-box TFs show significantly enriched binding of T-box TFs to promoter-proximal and intermediate upstream elements. Deletion analysis of the Xbra target *fgf4* suggests that both binding locations are required for appropriate gene expression (Casey et al., 1998). Our experiments indicate that promoter-proximal binding may be important for T-box TFs to recruit RNAPII and induce mesoderm-specific transcription. Recent work in the *Drosophila* embryo emphasizes the importance of promoters in recruiting RNAPII paused for robust and tissue-specific gene expression (Lagha et al., 2013). The patterns of DNA occupancy and differential expression analyses reveal T-box TF regulatory inputs that define neuromesodermal bipotency and prime mesoderm for further differentiation as follows: (1) stem cell maintenance by autoregulation of *Xbra*/*Xbra3* via fibroblast growth factor (FGF) signaling (Schulte-Merker and Smith, 1995) and regulation of retinoic acid levels by cyp26a1 (Martin and Kimelman, 2010) beyond the tail bud stage; (2) specification of paraxial mesoderm by *Tbx6* (Chapman and Papaioannou, 1998), *msgn1* (Wittler et al., 2007), and *foxc1* (Wilm et al., 2004); (3) myogenic differentiation by *myf5*, *myoD*, *myos*, and *actc1*; (4) patterning of presomitic mesoderm by *delta2* (Jen et al., 1997), *esr4*, *esr5* (Jen et al., 1999), *LOC773709* (this study), *mespa*, *mespb* (Sparrow et al., 1998), *mesp2*, *ripply2.1*, and *ripply2.2* (Kawamura et al., 2005; Kondow et al., 2006); and (5) continuous protection from neuralization by repression of neurogenic genes, such as *sox3* and *pax3*. Interestingly, these neural markers show as much high-density T-box TF binding as some activated target genes mentioned above. These binding clusters may define neuromesodermal bipotency, as they have recently been defined as superenhancers conferring cell identity (Whyte et al., 2013). Overexpression of *sox3* and *pax3* in anatomical positions of the chordoneural hinge and the posterior wall of the neurenteric canal reflects the local shift of neuromesodermal identity in T-box TF-depleted embryos, such that the loss of axial and paraxial mesoderm is accompanied by the gain of ventrolateral and dorsal neural tissue, respectively (Figure 7Biii).

The way in which T-box TFs suppress transcription of neurogenic genes is not known, although protein phosphorylation (Hwang et al., 2005) and corecruitment of a repressor complex (Kawamura et al., 2008) might both be involved in turning these activators into repressors. Indirect repression via T-box TF-dependent signaling pathways, such as FGF, retinoic acid, and Wnt, may also be involved in determining the fate of neuromesodermal stem cells (Li and Storey, 2011; Olivera-Martinez et al., 2012). Notably, some mesoderm-specific targets, such as *mespa* and *ripply2.1*, are only activated in presomitic mesoderm (S-I/S-II), where expression of Eomes, VegT, and Xbra3 is low or virtually absent. It is possible that these T-box TFs act as “placeholders” for Tbx6, which continues to be expressed in newly emerging paraxial (presomitic) mesoderm and can activate *ripply2.1* expression (Hitachi et al., 2009; Figure 7Bii). Interestingly, presomitic mesoderm retains neuromesodermal plasticity, because Tbx6 mutant mice form two supernumerary neural tubes at the expense of paraxial mesoderm (Chapman and Papaioannou, 1998). In contrast, Eomes, VegT, and Brachyury define neuromesodermal bipotency at an earlier stage, and thus their loss leads to one oversized neural tube.

Together, our experiments demonstrate that a T-box TF collective controls the emergence and fates of the bipotential neuromesodermal stem cells at the caudal end of the vertebrate embryo. The presence of these TFs causes cells to differentiate as mesoderm, and their absence permits them to fulfill their neural potential. Our work provides mechanistic insights into the way in which T-box TFs act together to regulate neuromesodermal fate, and this will inform attempts to define the differentiation pathways of embryonic and induced pluripotent stem cells.

## Experimental Procedures

### Embryo Culture

In vitro-fertilized *X. tropicalis* and *X. laevis* embryos were cultured in 5% Marc’s modified Ringer (MMR) at 20°C–28°C or 10% normal amphibian medium (NAM) at 14°C–25°C, respectively. Embryos were staged according to Nieuwkoop and Faber (1994). For details on knockdown and (hormone-inducible) overexpression experiments, see below and the Extended Experimental Procedures. All *Xenopus* studies complied fully with the UK Animals (Scientific Procedures) Act 1986 as implemented by the University of Cambridge and the MRC National Institute for Medical Research.

### Dexamethasone-Inducible GR Assays

For animal cap assays, *X. laevis* embryos were injected with 400 pg RNA encoding Xbra-GR (Tada et al., 1997), VegT-GR (White et al., 2002), and/or Eomes-GR (Extended Experimental Procedure). Animal caps were dissected at the blastula stage and cultured in 75% NAM at 20°C until sibling embryos reached stage 10.5. Half of the control and injected caps were then preincubated for 30 min in 10 μM chx and then left untreated or treated with 2 μM dexamethasone (dex), 10 μM chx, or both for about 3 hr until sibling embryos reached stage 12.5. We used a similar experimental set-up for the rescue of *msgn1* transcription in Xbra/Xbra3-depleted embryos (Extended Experimental Procedures). Animal caps and embryos without dex and/or chx treatments were incubated with dex and chx solvents ethanol and DMSO.

### Transcriptome-wide Single and Differential Expression Analysis

Total RNA to the amount of two *X. tropicalis* embryos (∼3 μg) was processed according to the TruSeq protocol (Illumina). Libraries were read paired-end along 55 bases on the HiSeq 2000 machine (Illumina). Bowtie 0.12.7 (Langmead, 2010) was used with the parameters -a–best -v 3 -y -I 0 -X 10000 to align the reads to the Ensembl JGI4.1 transcriptome. Any read pair that aligned to multiple transcripts of different genes was discarded, and any read pair that mapped to one or many transcripts of the same gene was counted once. Quantitative readouts from RNA-seq experiments were analyzed with DESeq (Anders and Huber, 2010). To display the RNA-seq profile as a track on the University of California Santa Cruz (UCSC) genome browser, reads were mapped to the genome of *X. tropicalis* as outlined for ChIP-seq profiles. The maximal distance between each read pair was set to 100 kb to allow paired reads to map to the genome across large introns. Resultant compressed binary version of sequence alignment/map files were converted to the bedGraph format using the bedTool function genomeCoverageBed.

### WMISH

WMISH was carried out as described in Monsoro-Burq (2007) with digoxigenin-labeled probes (Extended Experimental Procedures). For sectioning, embryos were dehydrated and embedded in paraffin. Ten micrometer sections were counterstained with Nuclear Fast Red.

### ChIP

This protocol (Extended Experimental Procedures), designed to process whole *Xenopus* embryos (which also proved to be applicable to zebrafish embryos), evolved from Lee et al. (2006). Key changes were made to the removal of residual fixative and the extraction of crosslinked nuclei from embryos prior to sonication to facilitate solubilization and efficient shearing of chromatin.

### ChIP-seq Analysis

Sequencing reads were mapped to the *X. tropicalis* genome assembly JGI4.1 using CLC Bio Genomics Workbench default settings. Nonspecific and ambiguous matches were ignored. ChIP-seq peaks were identified using MACS 2.0.4 (Zhang et al., 2008). Genomic coordinates of peaks are summarized in Table S1. Binding (pile-up of reads in Figure 1A; peak p values in all other figures) and transcript profiles were visualized on the UCSC genome browser. The nearest genes to peaks were found by ranking distances between peak summits and TSS of Ensembl genes (JGI4.1) using MySQL 5.6.2. Homer (Heinz et al., 2010) was used to perform metagene analysis and create tag and motif density maps. R, Excel, Cluster3, and JavaTreeview were subsequently used to combine different ChIP/RNA-seq data sets and visualize data as histograms, Venn diagrams, or heat maps. De novo motif analysis was performed with cisFinder (Sharov and Ko, 2009). See Extended Experimental Procedures for further details.

### Surface Plasmon Resonance

The affinity of Xbra binding to different DNA motifs was measured on an Octet RED biolayer interferometer. Biotinylated DNA oligonucleotides (Extended Experimental Procedures) were immobilized on streptavidin biosensors at concentrations in the range 0.5–0.7 μg/ml. Binding of native Xbra protein (Extended Experimental Procedures) at concentrations of 3 nM to 3.8 μM was measured at 25°C in a 5–10 min association step. The buffer contained 10 mM sodium phosphate pH 7.4, 150 mM NaCl, 0.005% Tween-20, and 0.1 mg/ml BSA. The (relative) amount of Xbra bound to the sensors was calculated from the amplitude of the response at the end of each association step. Equilibrium dissociation constants (K_d_) were determined by fitting the response as a function of the Xbra concentration.

Extended Experimental Procedures **Capped mRNA and Morpholinos**

Capped mRNA was transcribed from linearized pCS2+ and pSP64T plasmid constructs (see below) using the mMessage mMachine kit (Ambion). Xbra and Xbra3 Morpholinos (MOs) were designed and synthesized by Gene Tools LLC: *Xbra* MO1, 5′-TGGAGAGACCCTGATCTTACCTTCC-3′; *Xbra* MO2, 5′-GGCTTCCAAGCGCACACACTGGG-3′; *Xbra3* MO1, 5′-GAAAGGTCCATATTCTCTTACCTTC-3′; *Xbra3* MO2, 5′-AGCTGTGCCTGTGCTCATTGTATTG-3′. MOs for zygotic *VegT* and *Eomes* were as described in Fukuda et al. (2010). A human *β-globin* MO was used as control. If necessary, injection samples were supplemented with human *β-globin* MO to match the total amount of MO used for multiple knock-downs.

**Constructs**

Eomes-GR was constructed as follows: the coding region of *Eomes* was amplified from pBluescript RN3/*Eomes* (gift from J. Gurdon) using *Pfu* polymerase (Stratagene) and primers with *Spe*I restriction sites (5′-CAGACTAGTATGGTGCCTGGCGCCTG-3′, 5′-CAGACTAGTAGAACTAGAGTAGAAAGAGTAATACCCAAG-3′). The *Spe*I-digested PCR product was cloned into the *Spe*I site of the vector pSP64T-GR (Tada et al., 1997) to fuse Eomes in frame to the C-terminal GR fragment.

N- and C-terminal 3xHA tagged Xbra3 constructs were created by Gateway cloning. The C-terminal version contains the last 22 bp of the 5′ UTR in addition to the coding sequence of *Xbra3* without stop codon. The N-terminal version contains two silent point mutations at the 5′ end of the full coding sequence to render the transcript unrecognizable to *Xbra3* MO2. *Xbra3* was amplified from IMAGE clone 5307982 using *Pfu* polymerase (Stratagene) and the following primers to introduce the amplicon unidirectionally into the entry vector pENTR/TOPO. N-terminal construct: 5′-CACCATGAGtACAGGaACAGCTG-3′, 5′-CTATAATGATGGAGGTGTCACAGA-3′. C-terminal construct: 5′-CACCCAGAAGAGGCATCAGCAATAC-3′, 5′-TAATGATGGAGGTGTCACAGAAG-3′. The entry vector constructs were recombined with N- and C-terminal 3xHA pCS2+ destination vectors to obtain final 3xHA tagged Xbra3 constructs.

For the production of native Xbra protein, Xbra was PCR-amplified (5′-CACAGATCTGAGAACCTCTACTTCCAGGGTATGAGTGCGACCGAGAGCTGCGCC-3′ including BglII and TEV site, 5′-CACGAATTCTTAGACTGATGGTGGCGCAACGGG-3′ including EcoRI site) from plasmid pSP64T-Xbra (Cunliffe and Smith, 1992) and inserted into the baculovirus transfer vector pBac2-His via BglII and EcoRI sites.

**Rescue Experiment with Dexamethasone-Inducible GR**

100 pg *Xbra-GR* RNA and 100 pg nuclear *lacZ* RNA (cell lineage tracer) were injected unilaterally into the lower marginal zone of blastomeres at the 4-cell stage. Early tailbud embryos (stage 20) were pre-incubated for 30 min in 35 μM chx before treating them with 10 μM dex and 35 μM chx or both for about 3 hr (embryos reaching stage 22-23) and fixing them for lacZ staining and WMISH.

**Total RNA Extraction and cDNA Synthesis**

RNA was extracted from *Xenopus* tissue using Trizol (Invitrogen) and subjected to two precipitations with ethanol and LiCl. 1-2 μg of total RNA was reverse transcribed with M-MLV RT (Promega) and random hexamers to create cDNA for diagnostic PCR or qPCR.

**Diagnostic PCR**

RedTaq polymerase (Sigma) was used for diagnostic PCR according to the manufacturer’s instructions. PCRs were run on a G-Storm GS1 thermo cycler. The splicing-blocking MO (MO1) of Xbra and Xbra3 were verified by diagnostic PCR and qPCR, respectively, using these primer sets (Figures S2A and S2B): *Xbra*, 5′-CGCAAAGAATGTGCAGTACC-3′, 5′-TTGTCAGCTGCCACAAAATC-3′. *Xbra3*, 5′-TGCAAGTTGGAAGTGAGAAGG-3′, 5′-GGAACCCATTCTCCATTGAC-3′. *odc1*, 5′-GCCATCGTGAAGACTCTCTCCC-3′, 5′-TTTGGGTGATTCCTTGCCAC-3′. The efficiency of disrupting correct splicing of *Xbra3* was quantified by qPCR taking the melting curves into account to discriminate wild-type from cryptic templates.

**qPCR**

Expression levels (RT-qPCR) or DNA enrichment (ChIP-qPCR) were quantified in real-time relative to gene or locus-specific standard curves by the LightCycler LC480 II (Roche). The LC480 II carried out 55 cycles of amplification with the following denaturing, annealing and elongation conditions: 10 s at 94°C, 60°C and 72°C respectively. qPCR runs were performed with 2-3 technical replicates. Differential expression analysis by RT-qPCR was normalized to the amount of total RNA (equivalent to one *X. tropicalis* embryo) because its yield from embryos is more consistent (+/− 10%, data not shown) over a wide range of developmental stages than the expression of house-keeping genes. p-values from a two-tailed Student’s t test were corrected for multiple hypothesis testing using the Benjamini-Hochberg procedure and indicated as FDRs. See Table S4 for primer sequences.

**WMISH Probes**

cDNA clone of interest was linearized by an appropriate restriction digest overnight at 37°C. The linearized plasmid was purified using the QIAquick PCR purification kit (QIAGEN). After in vitro transcription according to the manufacturer’s instruction (Roche) with the appropriate RNA polymerase (SP6, T3 or T7) to create and anti-sense (AS) or sense (SE), the DNA template was removed by treating the reaction with TurboDNase (Ambion). The RNA probe was purified by spinning it through a resin column (Chroma Spin-100 DEPC-water column, Clontech). Eventually, a 10x stock of the RNA probe was made by diluting it with hybridization buffer to 10 ng/μl and stored at −20°C. This hybridization buffer consisted of 50% formamide, 5x SSC, 1x Denhardt’s solution, 10mM EDTA, 1mg/ml Torula RNA, 100 μg/ml heparin, 0.1% Tween-20 and 0.1% (w/v) CHAPS.

The probes were generated as follows: *actc1* AS (0.4 kb) probe was transcribed using SP6 from EcoRI-linearized pSP21 (*actc1*) (Mohun et al., 1984). *aldh1a2* AS (1.6 kb) probe was transcribed using T7 from BamHI-linearized pCS2+ (*aldh1a2*) (Chen et al., 2001). *Xbra* AS (2.2 kb) probe was transcribed using T7 from BglII-linearized pSP73 (*Xbra*) (Smith et al., 1991). *cav1* AS (1.4 kb) probe was transcribed using T7 from BglII-linearized pExpress1 (*cav1*) (IMAGE: 7024946). *cyp26a1* AS (1.0 kb) probe was transcribed using T7 from BamHI-linearized pCR2.1-TOPO (*cyp26a1*) (this study, see below). *delta2* AS (2.9 kb) probe was transcribed using T3 from SalI-linearized pSP72 (*delta2*) (Jen et al., 1997). *eomes* AS (2.1 kb) probe was transcribed using T7 from BamHI-linearized pCRII-TOPO (*eomes*) (this study, see below). *esr5* AS (0.9 kb) probe was transcribed using T3 from SalI-linearized pBluescript KS- (*esr5*) (Sparrow et al., 1998). *fgf8* AS (0.8 kb) probe was transcribed using T3 from XbaI-linearized pBluescript (*fgf8*) (European *Xenopus* Resource Centre). *foxc1* AS (1.5 kb) probe was transcribed using T7 from EcoRI-linearized pExpress1 (*foxc1*) (IMAGE: 7977266). *gdf3* AS (1.5 kb) probe was transcribed using T7 from EcoRI-linearized pCS2+ (*gdf3*) (Sun et al., 1999). *hoxd8* AS (1.0 kb) probe was transcribed using T7 from HindIII-linearized pCR2.1-TOPO (*hoxd8*) (this study, see below). *LOC733709* AS (1.4 kb) probe was transcribed using T7 from BamHI-linearized pCS108 (*LOC733709*) (IMAGE: 7551946). *LOC733709* SE (1.4 kb) probe was transcribed using SP6 from NotI-linearized pCS108 (*LOC733709*) (IMAGE: 7551946). *mespa* AS (0.9 kb) probe was transcribed using T3 from XbaI-linearized pBluescript KS- (*mespa*) (Sparrow et al., 1998). *msgn1* AS (1.0 kb) probe was transcribed using T7 from EcoRI-linearized pCMV-Sport6.ccdB (*msgn1*) (IMAGE: 699331). *myf5* AS (1.2 kb) probe was transcribed using SP6 from BamHI-linearized pSP73 (*myf5*) (Hopwood et al., 1991). *myh1* AS (0.4 kb) probe was transcribed using SP6 from NcoI-linearized pGEM (*myh1*) (Vernon and Philpott, 2003). *myoD* AS (0.7 and 1.1 kb) probe was transcribed using T7 from XhoI- or PspOMI-linearized pBluescript (*myoD*) (Hopwood et al., 1989). *N-tubulin* AS (1.3 kb) probe was transcribed using T3 from NcoI-linearized pBluescript KS+ (*N-tubulin*) (Richter et al., 1988). *not* AS (1.1 kb) probe was transcribed using T7 from HindIII-linearized pCS2+ (*not*) (von Dassow et al., 1993). *pax3* AS (3.0 kb) probe was transcribed using T7 from SmaI-linearized pExpress1 (*pax3*) (IMAGE: 7981250). *sox3* AS (1.6 kb) probe was transcribed using T7 from SmaI-linearized pBluescript SK+ (*sox3*) (Zygar et al., 1998). *tal1* AS (1.3 kb) probe was transcribed using SP6 from XhoI-linearized pGEM-7Zf+ (*tal1*) (European *Xenopus* Resource Centre). *vegT* AS (1.2 kb) probe was transcribed using T7 from BamHI-linearized pCR2.1-TOPO (*vegT*) (this study, see below). *ventx2.1* AS (1.2 kb) probe was transcribed using T7 from EcoRI-linearized pBluescript SK- (*ventx2.1*) (Ladher et al., 1996). *wnt11* AS (2.2 kb) probe was transcribed using SP6 from XbaI-linearized pGEM-7Zf+ (*wnt11*) (Ku and Melton, 1993).

The DNA fragments for the *cyp26a1*, *hoxd8* and *vegT* WMISH probes were amplified from *X. tropicalis* cDNA mix from stage 11, 12 and 14 using Advantage 2 polymerase (Clontech) inserted into pCR2.1-TOPO by TOPO TA cloning and the following primer pairs:

**cyp26a1:**

forward, 5′-ATGCAAATCCCAGACACCAC-3′

reverse, 5′-AAGCCTTCGAGGAAATGACC-3′

**hoxd8:**

forward, 5′-CCGGTCCAATATCCTGACTG-3′

reverse, 5′-TCGAACCGTGGTTTACAATTC-3′

**vegT:**

forward, 5′-TTCAATGCATCGCTACAAGC-3′

reverse, 5′-TGCGTTAGCACACACTTTCC-3′

The WMISH probe for *Eomes* was created from pCRII-TOPO including the PCR amplicon used for making the Eomes-GR construct.

**ChIP**

The following antibodies were used for ChIP: mouse monoclonal (8WG16) to RNAPII (Abcam, ab817; Covance, MMS-126R), rabbit polyclonal to H2A.z (Abcam, ab4174), Eomes, VegT and Xbra (Stennard et al., 1999), as well as mouse and rabbit IgG (Santa Cruz, sc-2025 and sc-2027, respectively).

Dejellied embryos were harvested at the developmental stage of interest and fixed at room temperature with fresh formaldehyde (molecular biology grade; Sigma F-8775) at a final concentration of 1% (v/v). The fixation time was determined empirically according to the species and the developmental stage of the embryo to achieve efficient chromatin shearing and ChIP enrichment. Early developmental stages were fixed over a longer time period than later stages: *X. tropicalis* gastrula (stage 11-12.5) for 20-25 min and early tailbud (stage 19-20) for 15 min. Next, the fixative solution was aspirated and the embryos were quickly washed 3-4 times with the corresponding ice-cold amphibian media (0.01x MMR for *X. tropicalis* embryos and 0.1x NAM for *X. laevis*). This inconspicuous washing step turned out to be crucial for the extraction of a relatively pure population of nuclei from crosslinked tissue. Immediate quenching of formaldehyde with glycine without any previous washing rendered the purification of nuclei an impossible task. Even though glycine is commonly considered not to have any crosslinking capacity due to its monovalent nature, the formaldehyde-glycine (Schiff-base) adduct might further react with N-terminal amino groups or arginine residues (Metz et al., 2004). Batches of 250 embryos were equilibrated in ice-cold HEG solution (50 mM HEPES-KOH pH 7.5, 1 mM EDTA pH 8.0, 20% glycerol). Once the embryos settled, excess HEG was removed. Embryo batches were snap-frozen in liquid nitrogen and stored at −80°C for future use. The following method for extracting crosslinked chromatin worked best with 2 ml of solutions E1, E2 and E3 (Lee et al., 2006) per 100-150 *X. tropicalis* or 50-75 *X. laevis* embryos. Before their immediate use, all solutions were supplemented with protease inhibitor tablets (complete EDTA-free, Roche) and 0.2 mM PMSF. In addition, buffer E1 was supplemented with 1 mM DTT. All biological samples and solutions were kept on ice. Centrifugations were carried out at 3,500 rpm for minimal 2 min in a refrigerated (4°C) centrifuge. Snap-frozen embryos were homogenized with E1 (50 mM HEPES-KOH pH 7.5, 140 mM NaCl, 1 mM EDTA pH 8.0, 10% glycerol, 0.5% Igepal CA-630, 0.25% Triton X-100). After centrifugation, the supernatant and any lipids attached to the wall were aspirated. The pellet was re-suspended in E1 and kept on ice for 10 min. After centrifugation, the supernatant was aspirated and the pellet re-suspended in E2 (Tris pH 8.0, 200 mM NaCl, 1 mM EDTA pH 8.0, 0.5 mM EGTA pH 8.0). This step was repeated once before keeping the lysate on ice for 10 min. After centrifugation, the supernatant was aspirated and the pellet re-suspended in E3 (10 mM Tris pH 8.0, 200 mM NaCl, 1 mM EDTA pH 8.0, 0.5 mM EGTA pH 8.0, 0.1% (w/v) Na-deoxycholate, 0.5% (w/v) N-lauroylsarcosine). The anionic detergents deoxycholate and N-lauroylsarcosine in E3 facilitated the solubilisation of most remaining yolk platelets. Thus, an additional spin made it possible to obtain a fairly pure population of cross-linked nuclei (data not shown). The pellet of cross-linked nuclei was re-suspended in 100-400 μl of buffer E3. At this stage of the protocol, several extractions were pooled to reach a final volume of 400-1,500 μl of cross-linked nuclei isolated from approximately 1,500 embryos at the maximum. Chromatin was solubilised and sheared to fragments of 100-500 bp peaking around 200 bp (Figure S1A) in a conical polysterene tube on ice water using a Misonix Sonicator 3000 Ultrasonic Liquid Processor equipped with a tapered 1/16” (1.6 mm) microtip (8-12 cycles of 30 s shock waves at 9-12 Watt interrupted by 1 min pauses). The sonicated nuclear extract was spun at 20,000 g speed for 10 min at 4°C. The supernatant was transferred to a low-retention 1.5ml Eppendorf tube and kept at 4°C for use on the same or following day or snap-frozen in liquid nitrogen and stored at −80°C for later usage. A sample equivalent to 10-20 embryos was used to check whether and the shearing of chromatin was successful. The appropriate Dynabeads M-280 were blocked with PBS containing 0.1% (w/v) BSA and coupled to ChIP-grade antibody (or control IgG). For creating a ChIP-Seq library from Eomes, VegT and Xbra immunoprecipitated DNA, approximately 3,000 *X. tropicalis* embryos were harvested and fixed at gastrula stage 11-12.5. The following calculation was taken into account when performing this experiment. A late *Xenopus* gastrula consists of roughly 40,000 cells, of which about 10% express Eomes, VegT or Xbra. That means that about twelve million cells expressing Eomes, VegT or Xbra were used to create the ChIP-Seq library, which is at the lower margin of what most investigators currently use as starting material for a ChIP-Seq experiment when mapping site-specific TFs in cell lines (10-100 million cells per ChIP-Seq). Approximately 5-10 μg of Eomes, VegT or Xbra antibody was coupled to 100-200 μl of the appropriate Dynabeads M-280 for ChIP-Seq. The soluble nuclear extract was prepared for immunoprecipitation by adding 1/10 volume of 10% cold Triton X-100. A sample equivalent to 5% ChIP input was collected at this stage and stored at 4°C to be processed further once the ChIP sample was ready for the reversal of cross-links. The rest of the nuclear extract was combined with antibody-coupled beads and incubated overnight. The beads were washed extensively six (ChIP-qPCR) or ten (ChIP-Seq) times with pre-chilled RIPA buffer (50 mM HEPES-KOH pH 7.5, 500 mM LiCl, 1mM EDTA, 1% Igepal CA-630, 0.7% (w/v) Na-deoxycholate, sterile filtered) and twice in pre-chilled TEN (10 mM Tris pH 8.0, 1 mM EDTA, 150 NaCl) for 5-10 min. If several tubes of the same ChIP were in use, the samples were pooled at this stage. Bound chromatin was eluted from the beads with elution buffer (50 mM Tris pH 8.0, 1 mM EDTA pH 8.0, 1% SDS) in a thermomixer (65°C, 30 min, 1000 rpm). ChIP and input samples were supplemented with 1/20 volume of 5 M NaCl before incubating them for 6-15 hr at 65°C to reverse cross-links. One volume of TE pH 8.0 and 200 μg/ml of CONCERT RNase A (Invitrogen) was added to the samples for a 1-h incubation at 37°C. The samples were further treated with 200 μg/ml of proteinase K (Ambion) for 2-4 hr at 55°C. Eventually, the DNA fragments were purified by the use of the QIAquick PCR purification kit (QIAGEN) for qPCR or with phenol:chloroform:isoamylalcohol and precipitated in ethanol for sequencing. The air-dried DNA pellet was dissolved in 32 μl of molecular-grade water. The amount of DNA was quantified using a Qubit fluorometer (Invitrogen) following the manufacturer’s instructions. The yield of co-immunoprecipitated DNA was about 10 ng, which created robust libraries. The same of amount of input DNA was used to create a library whose mapped reads were subtracted from the genome-wide ChIP profile to account for any shearing bias and other experimental artifacts.

**Preparation and Sequencing of ChIP-seq Library**

The single-end library was generated based on the manufacturer’s instructions (Illumina) with minor modifications. DNA fragments were end-repaired, ligated to adaptors at a final dilution of 1:480, size-selected to 200-300 bp by E-gel (Invitrogen) and PCR (18 cycles) amplified. The DNA Clean & Concentrator-5 kit (Zymo Research) was used to clean up reactions. DNA was eluted from the column at 50°C. Upon solid phase reversible immobilisation (SPRI) purification, the integrity of the library was checked on the Agilent 2100 Bioanalyzer using an Agilent DNA 1000 chip. ChIP-Seq libraries were sequenced for 40 cycles on the Illumina Genome Analyzer_IIx_.

**ChIP-seq Analysis**

Gene co-ordinates (gene ID, scaffold, gene start and end, strand, gene description and symbol) were downloaded from the ensembl browser via BioMart as a tab-separated plain text file. The ensembl JGI4.1 gene table (scaffold, gene start, gene end, strand, description) was manually updated as follows: *esr5* (scaffold_1051, 54853, 55447, 1, LOC100135364), LOC733709 (scaffold_73, 993469, 995810, 1, LOC733709), ripply2.1 (scaffold_375, 3181, 5676, −1, CR760245), *ripply2.2* (scaffold_76, 3643, 9247, 1, CU025073) and *tbx6* (scaffold_1655, 34433, 36638, −1, MGC79458).

For metagene analysis of ChIP-Seq data, peak assignment was restricted to the nearest gene. The ensembl JGI4.1 exon and UTR table was manually updated as follows: *esr5* (exon start, 54853; exon end, 55447), *LOC733709* (exon start, 993469; exon end, 993562; 5′ UTR start, 993469; 5′ UTR end, 993498; exon start, 994385; exon end, 994565; exon start, 994745; exon end, 995520; 3′ UTR start, 994969; 3′ UTR end, 995520; exon start, 995531; exon end, 995810; 3′ UTR start, 995531; 3′ UTR end, 995810), *ripply2.1* (exon start, 3181; exon end, 3688; exon start, 4187; exon end, 4254; exon start, 4993; exon end, 5074; exon start, 5555; exon end, 5676), *ripply2.2* (exon start, 3643; exon end, 3795; exon start, 4739; exon end, 4802; exon start, 5477; exon end, 5594; exon start, 7696; exon end, 9247) and *tbx6* (exon start, 34433; exon end, 34683; exon start, 36471; exon end, 36638; 5′ UTR start, 36589; 5′ UTR end, 36638).

Peaks were divided into several groups depending on their localization relative to the nearest gene: > 5 kb (distal), 1-5 kb (intermediate) and 1 kb (proximal) upstream and downstream of the gene body as well as the gene body, untranslated regions and exons. Peaks within intronic regions were calculated by subtracting the number of peaks within exons from those within gene bodies. A histogram of distances was created by means of the functional language R with breaks of 200 bp ranging from 20 kb up- and downstream from gene bodies to 2 kb downstream and upstream from the TSS and TES, respectively. The frequency map of peaks was assembled in Excel and the *x* axis re-labeled in Adobe Illustrator. To quantify the degree and determine the identity of shared and non-shared target genes between two ChIP-Seq experiments, the lists of target genes were loaded into the same MySQL table. In MySQL database terminology, shared and non-shared target genes were found by the conventional joining and left joining of the table on itself, respectively. Left joining created a null entry for each gene not being detected in both ChIP-Seq experiments. Querying the table for subsequent null entries retrieves all genes that are unique to either ChIP-Seq experiment. Alternatively, the R ‘merge’ function was iteratively used to combine more than two ChIP-Seq experiments. Results were imported into excel and auto-filtered accordingly. To cluster and/or visualize genomic binding profiles centered on the transcription start site (TSS) of target genes, peaks p-value were binned to 400 bp intervals from the TSS to genomic regions 10 kb up- and downstream. Peaks beyond the distance of 10 kb were assigned to a single bin for visualization purposes only (see Figure S2F). For de novo motif discovery analysis summit co-ordinates obtained from the peak caller MACS were uploaded as a custom track into the UCSC genomic browser. Repeat-masked DNA sequences of 200 bp and 400 bp regions flanking the peak summit were retrieved from the UCSC table browser in FASTA format and imported into cisFinder (Sharov and Ko, 2009). 200 bp regions were screened for over-represented motifs under the following conditions. The FDR was set to 1%. Motifs were called when enriched at least 2-fold. Motifs were clustered according to similarity of their position-weight matrices (PWMs) with a match of at least 75%. Both strands were searched for motifs. The PWMs were improved based on the number of occurrences of motif clusters in the sequence file using the default settings. Frequency histograms were created in R from a list of co-ordinates representing the starts of each motif found within extended binding regions of 400 bp. These lists of co-ordinates were obtained by running the patternScan C script (Sharov and Ko, 2009). Command line tools were used to assemble motif parameters such as the PWM-matching score into a file of BED format. The score 1.5 to 5 was scaled to 1000 to display the corresponding motifs in shades of gray on the UCSC browser (Figure S1E). In addition, the co-ordinates were extended 5′ and 3′ by 40 bp to make the motifs discernible on the genome browser when stretched to several kilobases.

**Mann-Whitney U Test for Enriched Xbra Binding in Target Gene Subsets**

A one-tailed and independent two-group Mann-Whitney U test was used as a non-parametric test to compare two out of five sets of DNA occupancies at a time within pre-defined genomic regions relative to TSSs (see Table S3). These bins of DNA occupancies (sum -log [peak p-value]) were created for subsets of target genes, which showed differential expression (≥1.5-fold, FDR < 10%) based on the transcriptome-wide analysis of Xbra/Xbra3 double morphants at stage 32. We asked whether set 1 shows higher DNA occupancy levels than set 2 (a null hypothesis that set 1 shows equal or lower DNA occupancies than set 2). This test was carried out with and without zero DNA occupancies at gastrula and early tailbud stage.

**Recombinant Xbra Protein Production and Purification under Native Conditions**

The following protein production and purification was carried out by Generon (UK). The Xbra construct with a cleavable (TEV) N-terminal His_10_-tag was transfected into Sf9 cells along with linearized baculovirus DNA (OET-cathepsin deleted strain). The recombinant virus was amplified to a high titer stock in Sf9 cells. For protein production, one liter of Hi5 insect cells at 2x10^6^ cells/ml was infected with the recombinant virus. Cells were harvested 48 hr post-infection and resuspended in 50 mM sodium phosphate pH 8.0, 0.3 M NaCl, 1% Triton X-100, 10% glycerol and complete EDTA-free protease inhibitors (Roche). Imidazole was added to a final concentration of 5 mM. After 30 min on ice and two rounds of sonication at 20 s at 20% maximal power (Branson Sonifier), the homogenate was clarified by centrifugation at 17,000 rpm for 20 min at 4°C. Xbra-His_10_ was first purified on a Ni^2+^ column (Ni-NTA, QIAGEN) by mixing the supernatant with Ni^2+^ resin for 2 hr at 4°C and washing Ni^2+^ column with an imidazole gradient (10 to 50 mM) in the above buffer. Xbra-His_10_ was eluted in 250 mM imidazole buffer, dialysed against phosphate buffered saline overnight and concentrated using ultrafiltration spin column Vivaspin 6 MWCO 5,000 (Sartorius). The protein concentrate was applied to a HiLoad 16/60 Superdex 200 PG column connected to the AKTAxpress chromatography system (GE Healthcare) for further purification of Xbra-His_10_ through gel filtration in 25 mM sodium phosphate pH 8.0 and 150 mM NaCl.

**Oligonucleotides for Surface Plasmon Resonance**

Forward oligonucleotides 5′-modified with Biotin [Btn] and purified by HPLC were purchased from Sigma.

**v1:**

forward, 5′-[Btn]AAAAAAAATTTCACACCTTA −3′

reverse, 5′-TAAGGTGTGAAAT-3′

**v1^T2A^:**

forward, 5′-[Btn]AAAAAAAATT**A**CACACCTTA-3′

reverse, 5′-TAAGGTGTG**T**AAT-3′

**v1^C5G^:**

forward, 5′-[Btn]AAAAAAAATTTCA**G**ACCTTA-3′

reverse, 5′-TAAGGT**C**TGAAAT-3′

**v2:**

forward, 5′-[Btn]AAAAAAAATAATTAGCATATTA-3′

reverse, 5′-TAATATGCTAATTAT-3′

**v4:**

forward, 5′-[Btn]AAAAAAAATTTAGCATTATTATGTTATA-3′

reverse, 5′-TATAACATAATAATGCTAAAT−3′

**Western Blotting**

Embryonic tissue was homogenized and left on ice for 5 min in 5 μl/embryo of phospho-safe extraction buffer (Novagen) supplemented with 0.2 mM PMSF and complete EDTA-free protease inhibitors (Roche). The homogenate was mixed with 5 μl/embryo FREON to remove yolk before SDS-polyacrylamide gel electrophoresis and Western blotting. Primary antibodies: rabbit polyclonal anti-Xbra serum (Cunliffe and Smith, 1994) 1:2000, rabbit polyclonal anti-Xbra (Stennard et al., 1999) 1:4000, mouse monoclonal anti-α-tubulin (Sigma) 1:5000, mouse monoclonal anti-β-actin, peroxidase (Sigma) 1:1000, anti-HA, peroxidase (3F10, Roche) 1:3000. Secondary antibody: goat anti-rabbit IgG (H+L) peroxidase (Uptima) 1:2000.

**Whole-Mount Immunohistochemistry**

WMIHC was performed as described in Klymkowsky and Hanken (1991). Rabbit polyclonal Xbra anti-serum (Cunliffe and Smith, 1994) was used at 1:500 and peroxidase-conjugated anti-rabbit IgG at 1:700.

**TUNEL Assay**

TUNEL was essentially performed as described in Trindade et al. (2003). To induce apoptosis in vivo, embryos were treated for 4 hr in 35 μM cycloheximide at 23°C. As a positive control for the TUNEL assay fixed embryos were incubated in 0.1 U/μl Turbo DNase (Ambion) for 20 min at room temperature.

## Author Contributions

G.E.G. conceived the study and carried out most experiments and postsequencing analysis. N.D.L.O., P.P., M.W.B.T., and M.J.G. helped with the processing of sequencing data. T.F. contributed Brachyury binding data from human embryonic stem cell derivatives. J.C.S. and S.R.M. carried out whole-mount immunohistochemistry for Xbra protein and SPR, respectively. G.E.G. and J.C.S. wrote the manuscript.

## Accession Numbers

The Gene Expression Omnibus accession numbers of the ChIP-seq and RNA-seq datasets reported in this paper are GSE48560 and GSE48663.

## Supplemental Information

Table S1. Summary of Genomic Regions Bound by Eomes, VegT, and Xbra in *X. tropicalis* Embryos, Related to Figures 1 and 4 Excel spreadsheets contain genomic coordinates (genome assembly JGI4.1), pile-up values, fold of enrichment, p, and FDR (q) values for each peak called by MACS 2.0.4 (Zhang et al., 2008) with indicated settings in the header of each sheet named after the corresponding ChIP-seq experiments: Xbra at gastrula stage, Xbra 12 (1) and (2), and early tail bud stage, Xbra 20 as well as for Eomes and VegT at gastrula stage, Eomes 12, and VegT 12, respectively.

Table S2. Differential Expression Analysis of Xbra/Xbra3 Knockdown Embryos versus Control Embryos at Early Tadpole Stage, Related to Figure 2 Excel spreadsheet contains Xbra/Xbra3 knockdown (KD)-mediated changes of the Ensembl JGI4.1 transcriptome (fold change and its statistical significance) in early tadpoles (stage 32), which was determined by RNA-seq followed by postsequencing analysis with DESeq (Anders and Huber, 2010).

Table S3. Positively Regulated Target Genes Feature Enriched DNA Occupancy of Xbra at Promoter-Proximal and Intermediate Upstream Regions, Related to Figure 3 Statistical significance (p value) of enriched DNA occupancies of Xbra within indicated genomic regions was calculated for different sets of target genes (sum [−log p] ≥ 25) and two developmental stages (gastrula and early tail bud) by means of a one-tailed Mann-Whitney U test. Target genes were grouped according to their transcriptional misregulation (≥1.5-fold; FDR < 10%) upon Xbra/Xbra3 KD at stage 32 (early tadpole). All target genes (sum [−log p] ≥ 25) as well as subsets of them without up- or downregulated genes, respectively, were used as controls. The second row of each developmental stage contains p values calculated from datasets from which no (zero) DNA occupancies were excluded. By means of this test, we asked whether set 1 (ordinate) shows higher DNA occupancy levels than set 2 (abscissa). See Extended Experimental Procedures for further details.

Table S4. RT-qPCR and ChIP-qPCR Primer Sequences, Related to Experimental Procedures RT-qPCR and ChIP-qPCR primers were designed by means of the web portal Primer3 to have an annealing temperature of 60°C and amplify specific products of 75 to 125 bp. If possible, RT-qPCR primers were chosen to span an intron longer than 500 bp or overlap the splice junction in mRNA transcripts to prevent amplification from any residual genomic DNA. ChIP-qPCR primers are labeled according to the name of the most proximal gene followed by the position relative to the transcription start site.

Document S1. Article plus Supplemental Information
