# Supplementary material for: In Vivo T-Box Transcription Factor Profiling Reveals Joint Regulation of Embryonic Neuromesodermal Bipotency
Source: Cell Rep. 2013 Sep 26;4(6):1185–96. doi: 10.1016/j.celrep.2013.08.012 (PMC3791401; doi:10.1016/j.celrep.2013.08.012)
Supplement: Table S3. Positively Regulated Target Genes Feature Enriched DNA Occupancy of Xbra at Promoter-Proximal and Intermediate Upstream Regions, Related to Figure 3 — Statistical significance (p value) of enriched DNA occupancies of Xbra within indicated genomic regions was calculated for different sets of target genes (sum [−log p] ≥ 25) and two developmental stages (gastrula and early tail bud) by means of a one-tailed Mann-Whitney U test. Target genes were grouped according to their transcriptional misregulation (≥1.5-fold; FDR < 10%) upon Xbra/Xbra3 KD at stage 32 (early tadpole). All target genes (sum [−log p] ≥ 25) as well as subsets of them without up- or downregulated genes, respectively, were used as controls. The second row of each developmental stage contains p values calculated from datasets from which no (zero) DNA occupancies were excluded. By means of this test, we asked whether set 1 (ordinate) shows higher DNA occupancy levels than set 2 (abscissa). See Extended Experimental Procedures for further details. [file mmc3.pdf]

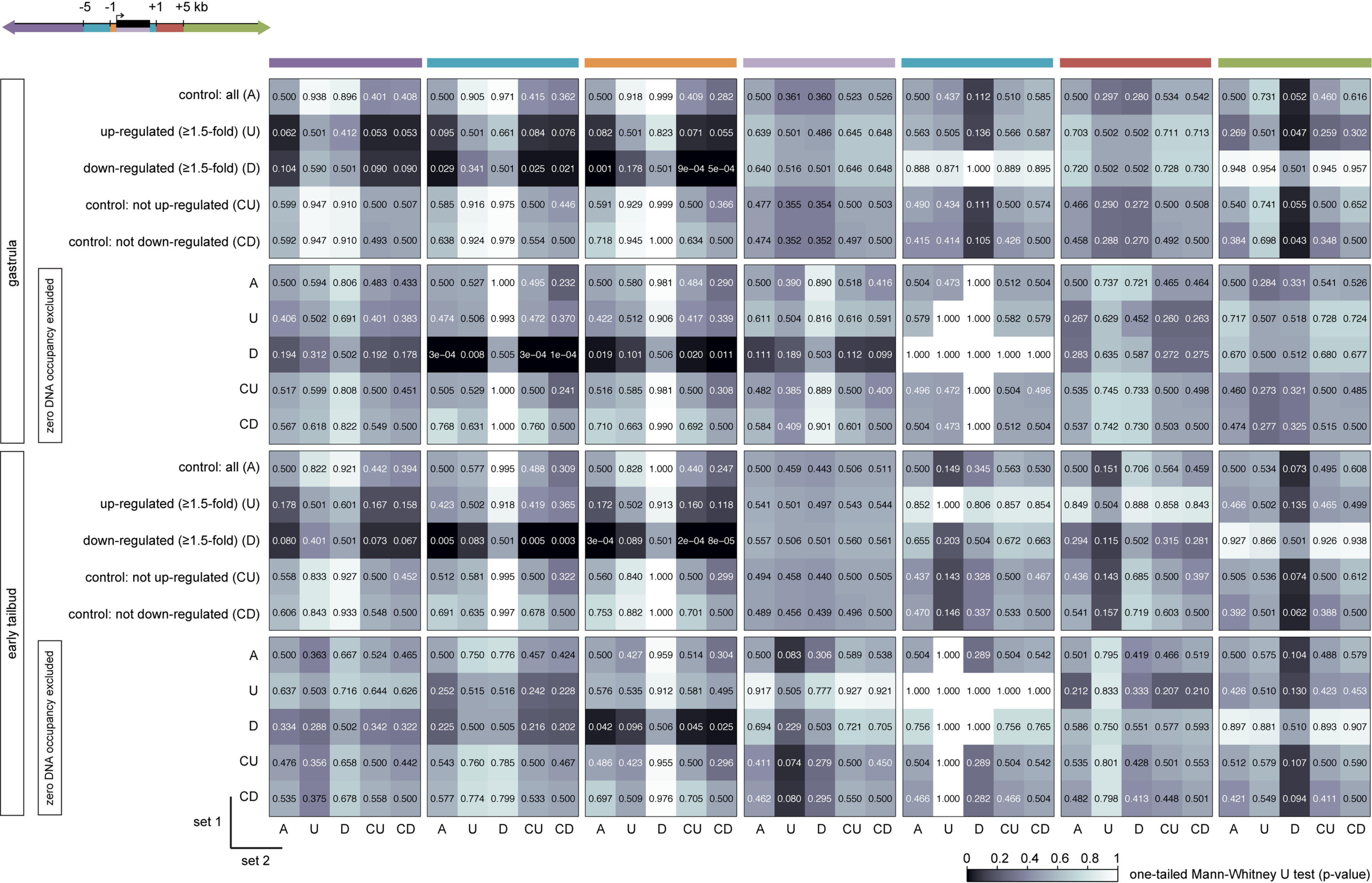
-5 -1 +1 +5 kb
gastrula
zero DNA occupancy excluded
control: all (A) 0.500 0.938 0.896 0.401 0.408 0.500 0.905 0.971 0.415 0.362 0.500 0.918 0.999 0.409 0.282 0.500 0.361 0.360 0.523 0.526 0.500 0.437 0.112 0.510 0.585 0.500 0.297 0.280 0.534 0.542 0.500 0.731 0.052 0.460 0.616
up-regulated (≥1.5-fold) (U) 0.062 0.501 0.412 0.053 0.053 0.095 0.501 0.661 0.084 0.076 0.082 0.501 0.823 0.071 0.055 0.639 0.501 0.486 0.645 0.648 0.563 0.505 0.136 0.566 0.587 0.703 0.502 0.502 0.711 0.713 0.269 0.501 0.047 0.259 0.302
down-regulated (≥1.5-fold) (D) 0.104 0.590 0.501 0.090 0.090 0.029 0.341 0.501 0.025 0.021 0.001 0.178 0.501 9e−04 5e−04 0.640 0.516 0.501 0.646 0.648 0.888 0.871 1.000 0.889 0.895 0.720 0.502 0.502 0.728 0.730 0.948 0.954 0.501 0.945 0.957
control: not up-regulated (CU) 0.599 0.947 0.910 0.500 0.507 0.585 0.916 0.975 0.500 0.446 0.591 0.929 0.999 0.500 0.366 0.477 0.355 0.354 0.500 0.503 0.490 0.434 0.111 0.500 0.574 0.466 0.290 0.272 0.500 0.508 0.540 0.741 0.055 0.500 0.652
control: not down-regulated (CD) 0.592 0.947 0.910 0.493 0.500 0.638 0.924 0.979 0.554 0.500 0.718 0.945 1.000 0.634 0.500 0.474 0.352 0.352 0.497 0.500 0.415 0.414 0.105 0.426 0.500 0.458 0.288 0.270 0.492 0.500 0.384 0.698 0.043 0.348 0.500
A 0.500 0.594 0.806 0.483 0.433 0.500 0.527 1.000 0.495 0.232 0.500 0.580 0.981 0.484 0.290 0.500 0.390 0.890 0.518 0.416 0.504 0.473 1.000 0.512 0.504 0.500 0.737 0.721 0.465 0.464 0.500 0.284 0.331 0.541 0.526
U 0.406 0.502 0.691 0.401 0.383 0.474 0.506 0.993 0.472 0.370 0.422 0.512 0.906 0.417 0.339 0.611 0.504 0.816 0.616 0.591 0.579 1.000 1.000 0.582 0.579 0.267 0.629 0.452 0.260 0.263 0.717 0.507 0.518 0.728 0.724
D 0.194 0.312 0.502 0.192 0.178 3e−04 0.008 0.505 3e−04 1e−04 0.019 0.101 0.506 0.020 0.011 0.111 0.189 0.503 0.112 0.099 1.000 1.000 1.000 1.000 1.000 0.283 0.635 0.587 0.272 0.275 0.670 0.500 0.512 0.680 0.677
CU 0.517 0.599 0.808 0.500 0.451 0.505 0.529 1.000 0.500 0.241 0.516 0.585 0.981 0.500 0.308 0.482 0.385 0.889 0.500 0.400 0.496 0.472 1.000 0.504 0.496 0.535 0.745 0.733 0.500 0.498 0.460 0.273 0.321 0.500 0.485
CD 0.567 0.618 0.822 0.549 0.500 0.768 0.631 1.000 0.760 0.500 0.710 0.663 0.990 0.692 0.500 0.584 0.409 0.901 0.601 0.500 0.504 0.473 1.000 0.512 0.504 0.537 0.742 0.730 0.503 0.500 0.474 0.277 0.325 0.515 0.500
early tailbud
zero DNA occupancy excluded
control: all (A) 0.500 0.822 0.921 0.442 0.394 0.500 0.577 0.995 0.488 0.309 0.500 0.828 1.000 0.440 0.247 0.500 0.459 0.443 0.506 0.511 0.500 0.149 0.345 0.563 0.530 0.500 0.151 0.706 0.564 0.459 0.500 0.534 0.073 0.495 0.608
up-regulated (≥1.5-fold) (U) 0.178 0.501 0.601 0.167 0.158 0.423 0.502 0.918 0.419 0.365 0.172 0.502 0.913 0.160 0.118 0.541 0.501 0.497 0.543 0.544 0.852 1.000 0.806 0.857 0.854 0.849 0.504 0.888 0.858 0.843 0.466 0.502 0.135 0.465 0.499
down-regulated (≥1.5-fold) (D) 0.080 0.401 0.501 0.073 0.067 0.005 0.083 0.501 0.005 0.003 3e−04 0.089 0.501 2e−04 8e−05 0.557 0.506 0.501 0.560 0.561 0.655 0.203 0.504 0.672 0.663 0.294 0.115 0.502 0.315 0.281 0.927 0.866 0.501 0.926 0.938
control: not up-regulated (CU) 0.558 0.833 0.927 0.500 0.452 0.512 0.581 0.995 0.500 0.322 0.560 0.840 1.000 0.500 0.299 0.494 0.458 0.440 0.500 0.505 0.437 0.143 0.328 0.500 0.467 0.436 0.143 0.685 0.500 0.397 0.505 0.536 0.074 0.500 0.612
control: not down-regulated (CD) 0.606 0.843 0.933 0.548 0.500 0.691 0.635 0.997 0.678 0.500 0.753 0.882 1.000 0.701 0.500 0.489 0.456 0.439 0.496 0.500 0.470 0.146 0.337 0.533 0.500 0.541 0.157 0.719 0.603 0.500 0.392 0.501 0.062 0.388 0.500
A 0.500 0.363 0.667 0.524 0.465 0.500 0.750 0.776 0.457 0.424 0.500 0.427 0.959 0.514 0.304 0.500 0.083 0.306 0.589 0.538 0.504 1.000 0.289 0.504 0.542 0.501 0.795 0.419 0.466 0.519 0.500 0.575 0.104 0.488 0.579
U 0.637 0.503 0.716 0.644 0.626 0.252 0.515 0.516 0.242 0.228 0.576 0.535 0.912 0.581 0.495 0.917 0.505 0.777 0.927 0.921 1.000 1.000 1.000 1.000 1.000 0.212 0.833 0.333 0.207 0.210 0.426 0.510 0.130 0.423 0.453
D 0.334 0.288 0.502 0.342 0.322 0.225 0.500 0.505 0.216 0.202 0.042 0.096 0.506 0.045 0.025 0.694 0.229 0.503 0.721 0.705 0.756 1.000 1.000 0.756 0.765 0.586 0.750 0.551 0.577 0.593 0.897 0.881 0.510 0.893 0.907
CU 0.476 0.356 0.658 0.500 0.442 0.543 0.760 0.785 0.500 0.467 0.486 0.423 0.955 0.500 0.296 0.411 0.074 0.279 0.500 0.450 0.504 1.000 0.289 0.504 0.542 0.535 0.801 0.428 0.501 0.553 0.512 0.579 0.107 0.500 0.590
CD 0.535 0.375 0.678 0.558 0.500 0.577 0.774 0.799 0.533 0.500 0.697 0.509 0.976 0.705 0.500 0.462 0.080 0.295 0.550 0.500 0.466 1.000 0.282 0.466 0.504 0.482 0.798 0.413 0.448 0.501 0.421 0.549 0.094 0.411 0.500
set 1
set 2
A U D CU CD A U D CU CD A U D CU CD A U D CU CD A U D CU CD A U D CU CD A U D CU CD
0 0.2 0.4 0.6 0.8 1
one-tailed Mann-Whitney U test (p-value)
